# Co-infection of tick-borne bacterial pathogens in ticks in Inner Mongolia, China

**DOI:** 10.1371/journal.pntd.0011121

**Published:** 2023-03-09

**Authors:** Dan Liu, Wulantuya, Hongxia Fan, Xiaona Li, Fangchao Li, Ting Gao, Xuhong Yin, Zitong Zhang, Minzhi Cao, Hiroki Kawabata, Kozue Sato, Norio Ohashi, Shuji Ando, Gaowa

**Affiliations:** 1 Inner Mongolia Key Laboratory of Tick-borne Zoonotic Infectious Disease, Department of Medicine, College of Hetao, Bayan Nur city, Inner Mongolia Autonomous Region, China; 2 Department of Bacteriology-I, National Institute of Infectious Diseases, Shinjuku-ku, Tokyo, Japan; 3 Laboratory of Microbiology, Department of Food and Nutritional Sciences, University of Shizuoka, Shizuoka, Japan; 4 Department of Virology-I, National Institute of Infectious Diseases, Tokyo, Japan; KU Leuven, BELGIUM

## Abstract

Tick-borne infectious diseases pose a serious health threat in certain regions of the world. Emerging infectious diseases caused by novel tick-borne pathogens have been reported that are causing particular concern. Several tick-borne diseases often coexist in the same foci, and a single vector tick can transmit two or more pathogens at the same time, which greatly increases the probability of co-infection in host animals and humans and can lead to an epidemic of tick-borne disease. The lack of epidemiological data and information on the specific clinical symptoms related to co-infection with tick-borne pathogens means that it is not currently possible to accurately and rapidly distinguish between a single pathogen infection and co-infection with multiple pathogens, which can have serious consequences. Inner Mongolia in the north of China is endemic for tick-borne infectious diseases, especially in the eastern forest region. Previous studies have found that more than 10% of co-infections were in host-seeking ticks. However, the lack of data on the specific types of co-infection with pathogens makes clinical treatment difficult. In our study, we present data on the co-infection types and the differences in co-infection among different ecological regions through genetic analysis of tick samples collected throughout Inner Mongolia. Our findings may aid clinicians in the diagnosis of concomitant tick-borne infectious diseases.

## Author summary

Ticks carry and transmit a variety of pathogens, and their host animals are widely distributed in nature with diverse epidemic links, so that several tick-borne diseases can coexist in the same foci, and even one type of tick or one tick can carry a variety of pathogens at the same time, these factors pose a serious public health threat. In our study, we summarized the regional differences in tick infection and co-infection rates outcomes in all Inner Mongolia of China, and found that pathogen diversity varied with ecogeographic differences such as gobi desert, grassland and forest. A variety of tick-borne bacterial pathogens were identified, with an overall high prevalence rate (61.4% of ticks infected), among infected ticks, 24.3% were co-infected. An unexpected high infection and co-infection rates of ticks collected from the Forest region of eastern Inner Mongolia (sample area 1) was detected. Geographical differences affect tick species and tick-borne pathogens, so the potential threat to humans or animals is also different. In particular, that that knowledge of co-infections is important as they do not just present diagnostic challenges, but the pathogens might play different roles within their respective hosts, thus modulating disease severity.

## Introduction

Tick-borne pathogens are transmitted via hematophagous blood-sucking ticks to hosts (including humans), in which they may cause infectious disease. In some cases, ticks harbor multiple pathogens, which can result in co-infection. The two forms of co-infection are interspecific infection and intraspecific infection with different genospecies, and regional differences between these two types of infection have been reported [1–3]. Distinct environmental conditions provide the habitat for specific tick species and several tick-borne diseases often coexist in the same foci, which defines their geographical distribution and, consequently, the areas of risk for human tick-borne infections [4–6]. In these areas, the probability of co-infection of host animals and humans is greatly increased, leading to an epidemic of tick-borne disease.

During the 20th century, Mitchell and colleagues proposed serological evidence of co-infection with *Borrelia burgdorferi*, *Babesia*, and human granulocytic *Ehrlichia* species in residents of Wisconsin and Minnesota in the USA [7]. In a 4-year prospective study conducted in Germany and Latvia, 75 of 192 patients (39%) were co-infected with tick-borne pathogens, and 61 of the 75 patients were co-infected with *B*. *burgdorferi* and *Babesia*, with a co-infection rate of 81% in *Ixodes ricinus* ticks [8]. Dibernardo and colleagues reported co-infection of *B*. *burgdorferi* and *Anaplasma phagocytophilum* in *Ixodes scapularis* ticks collected in Canada [9]. These studies suggested that co-infection of *B*. *burgdorferi* with *Babesia* is common in both patients and tick samples. Lu and coworkers also found *Candidatus* R. tarasevichiae infection in patients with severe fever that were also infected with thrombocytopenia syndrome virus, with a co-infection rate of 9.4% (77/823) in China [10]. The results of laboratory examination and clinical manifestations suggested that the co-infection group included more severe cases than the single infection group. Furthermore, the course of disease was longer, the recovery of laboratory indicators was slower, and fatalities were reported among the co-infection group [10,11].

Located on the border of China and Russia, the Greater Hinggan Mountains in the eastern part of Inner Mongolia are rich in wildlife and have a diverse ecosystem. This region is one of the major epidemic areas of tick-borne infectious diseases in China because its unique geographical and ecological features make it an ideal habitat for ticks [3,12–14]. In Inner Mongolia, and elsewhere in China and the rest of the world, limited research has been carried out on the occurrence of different genospecies in co-infections in host-questing ticks, despite progress on tick-borne infections. Indeed, most studies have focused on the identification of diversity in a single or a few pathogens, or on the prevalence of pathogen species [15,16]. Our present study aimed to detect the co-infection rates and co-infection diversity of tick-borne pathogens in questing ticks collected from three different ecological sites in Inner Mongolia.

## Materials and methods

### Ethics statement

The collection of ticks from the body surface of cattle, goats, and horses in this study was verbally approved by the animal owners and performed in strict accordance with the National Guidelines for Experimental Animal Welfare of China (2006–398). In addition, it has been applied and reviewed by the Animal Experimental Ethics Committee of the Medical Department of Hetao College, and it has been put on record in accordance with the "Animal Experimental Ethics Review Measures of Hetao College" ([2022] No. 112).

### Study area and tick sampling sites

**Fig 1:**
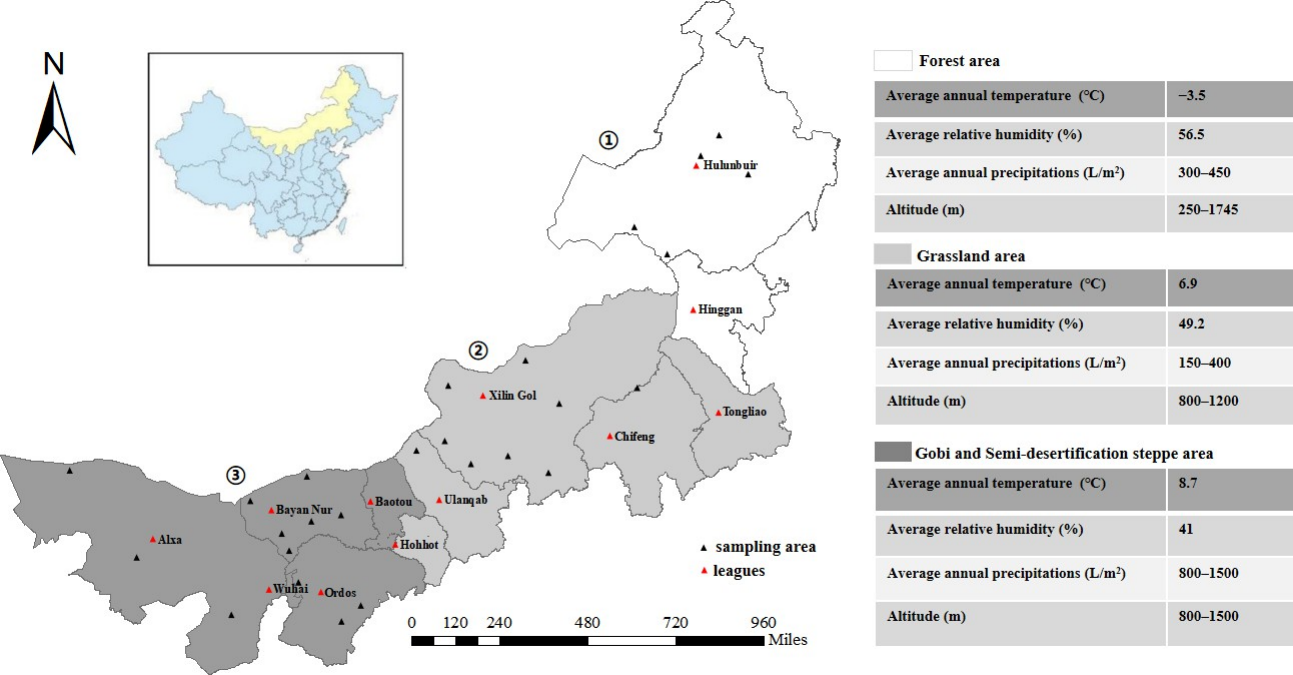
Sampling areas. Geographical areas used for sampling were located in the north of China, and were divided mainly on the basis of different ecological and environmental characteristics. Sampling area 1 was a mainly forested habitat, sampling area 2 was a grassland habitat, and sampling area 3 was the semi-desertification steppe / Gobi Desert. The tick sampling collection period was from May 2015 until June 2019. ①—Sampling area 1 covering two leagues: Hulunbuir and Hinggan. ②—Sampling area 2 including Hohhot, Ulanqab and Xilin Gol, Tongliao and Chifeng. ③—Sampling area 3 including Baotou, Wuhai, Ordos, Bayan Nur, and Alxa. Map source: National Earth System Science Data Center (http://www.geodata.cn/data/datadetails.html?dataguid=223718677040067&docid=4590).

Inner Mongolia Autonomous Region is located in the northern frontier of China (97°E–126°E; 37°N–53°N), bordering Mongolia and Russia 4200 km to the north. The natural grassland in Inner Mongolia is vast and broad, with the total area ranking highest among the five grasslands in China, and it is an important region for livestock production. The topography of Inner Mongolia comprises mainly the Mongolian plateau, which is complex and diverse in form, with an average elevation of about 1000 m and a temperature that changes greatly between winter and summer. The sampling sites for this study were distributed across 103 counties (banners) of 12 league sites, covering three ecologically and geographically distinct areas of Inner Mongolia (Fig 1).

From March to September of each year from 2015 to 2019, ticks were collected from vegetation in the forest by the lab-cloth flagging method from area 1, and exoparasitic ticks were collected from cattle, goat, sheep, camel and horse by the animal physical examination method in area 2 and 3. Each area represented a distinct habitat. In sampling area 1, a forested area in the north-eastern part of Inner Mongolia, the main habitat was primeval forest at an altitude of 250–1745 m, with an average annual temperature of ˗3.5°C, and annual precipitation of 300–450 mm. In sampling area 2, a grassland area in central Inner Mongolia, the habitat is considered a frigid temperate zone of semi damp grassland with monsoonal conditions, at an altitude of 800–1200 m, and with annual precipitation of 150–400 mm. In sampling area 3, an area encompassing the Gobi Desert and the semi-desertification steppe of the western part of Inner Mongolia, the land is arid, at an altitude of 800–1500 m, and with annual precipitation of 40–240 mm (Fig 1).

Land cover data of Inner Mongolia were obtained free from the National Earth System Science Data Sharing Infrastructure (http://www.geodata.cn). ArcGIS 10.2 software was used for visualization.

### Tick species identification, DNA extraction, and detection

Ticks were identified by morphological characteristics combined with tick *mt-rrs* gene identification method [17]. The ticks were soaked with sodium hypophosphite, 75% ethanol, and iodophor for 5 min, then washed with sterile water, dried naturally, and DNA was extracted using a genomic extraction kit (Qiagen, Hilden, Germany). Extracted DNA was stored at ˗20°C before use.

### Detection of co-infections with other tick-borne pathogens

PCR was used for detection of the citrate synthase gene (*gltA*) in spotted fever group rickettsiae (SFGR). The *gltA*-positive samples were classified, and representative samples were sequenced according to the tick species and regional distribution. The *Rickettsia* outer membrane protein A gene (*rOmpA*) was also amplified for confirmation of the *gltA* PCR [18]. The outer membrane protein-1 gene (*p28*/*omp-1*) of *Ehrlichia* and the major surface protein-2 gene (*p44*/*msp2*) of *Anaplasma* were detected by nested PCR [12,19]. Targeting the *16S rRNA* gene for borreliae, DNA primers and Taqman probes were designed from conserved sequences. Specific DNA probes were labeled with two types of fluorescent dyes, 6-carboxyfluorescein (FAM) and 4,7,2′-trichloro-7′-phenyl-6-carboxyfluorescein (VIC), and were conjugated with the non-fluorescent quencher (NFQ) and minor groove-binder architectural protein (MGB) according to a report by Barbour and colleagues (Table 1) [20]. Multiplex PCR was performed by real-time PCR according to a previously described protocol [17]. The 16S rRNA PCR-positive samples were classified, and conventional PCRs based on the borrelial flagellin (*flaB*) gene or the glycerophosphodiester phosphodiesterase (*glpQ*) gene were performed for confirmation of the real-time PCR results [21]. The PCR-specific primers and reaction conditions were derived from our previous studies [12,18–21], and the primers used in these experiments were synthesized by Nanjing Kingsley Biotechnology Company (Nanjing, China).

**Table 1:** DNA primers used in this study.

Target group	Target gene	Primer	Sequences(5’→3’)	Reference
SFGR*^1^	*gltA*	*gltA*-F	CGAACTTACCGCTATTAGAATG	[17]
		*gltA*-R	CTTTAAGAGCGATAGCTTCAAG	
	*rOmpA*	*rOmpA*-F	TGGTGGAGCTCATAAGTTACA	[12]
		*rOmpA*-R	AGTTACATTTCCTGCACCTAC	
*Ehrlichia*	*P28/omp1*	conP28-F1	AT[C/T]AGTG[G/C]AAA[A/G]TA[T/C][A/G]T[G/A]CCAA	[18]
		conP28-R1	CAATGG[A/G][T/A]GG[T/C]CC[A/C]AGA[A/G]TAG	
		conP28-F2	TTA[G/A]AA[A/G]G[C/T]AAA[C/T]CT[T/G]CCTCC	
		conP28-R2	TTCC[T/C]TG[A/G]TA[A/G]G[A/C]AA[T/G]TTTAGG	
*Anaplasma*	*P44/msp2*	p3726	GCTAAGGAGTTAGCTTATGA	[18]
		p3761	CTGCTCT[T/G]GCCAA(AG)ACCTC	
		p4183	CAATAGT[C/T]TTAGCTAGTAACC	
		p4257	AGAAGATCATAACAAGCATTG	
*Borrelia*	*flaB*	*flaB*-F	GCTGAAGAGCTTGGAATGCAACC	[19]
		*flaB*-R	TGATCAGTTATCATTCTAATAGCA	
	*glpQ*	*glpQ*-F	CATACGCTTATGCYTTRGGMGCTGA	
		*glpQ*-R	GCAACCTCTGYCATACCTTCTTSTG	
	16S rRNA	16S_RT_F	GCTGTAAACGATGCACACTTGGT	[20]
		16S _RT_R	GGCGGCACACTTAACACGTTAG	
		BB_FAM	FAM-TTCGGTACTAACTTTTAGTTAA-NFQ-MGB	
		BM_VIC	VIC-CGGTACTAACCTTTCGATTA-NFQ-MGB	

*1: SFGR (Spotted fever group rickettsiae).

### PCR product purification and sequence analysis

The obtained PCR products of *Anaplasma* and *Ehrlichia* were gel-purified and cloned into a pCR2.1 vector using a TA Cloning kit (Thermo Fisher Scientific, Waltham, MA, USA). *Escherichia coli* DH5*α* (TOYOBO, Osaka, Japan) was transformed with the recombinant plasmids. Ten clones were selected randomly for each PCR product and the insert DNA of each clone was sequenced. Other PCR products were purified and sequenced directly. All obtained sequences were assembled and translated into protein sequences using the Sequencher program (Gene Codes Corp., Ann Arbor, MI, USA). Homology searches and species identification were performed using blastn or blastp (http://blast.ncbi.nlm.nih.gov/Blast.cgi). Phylogenetic analysis of the *flaB*, *glpQ*, *gltA*, *p28*/*omp-1*, and *p44*/*msp2* sequences were performed using MEGA 7 with 1000 bootstrap replicates [22].

### Statistical analysis

Excel software was used to establish the database and IBM SPSS Statistics version 19 (IBM R Corporation, Chicago, IL, USA) was used for data analysis. Data counts were described by the number of cases (percentage), and the chi-square test (*χ*^2^) was performed on data relating to the tick infection ratio of geographic groups. A *P*-value was determined to be statistically significant when *P* < 0.05.

## Results

### Tick collection

During the spring to summer seasons (April to July) of 2015 to 2019, a total of 6456 adult tick samples belonging to eight species and five genera were collected in three different ecological environment sampling areas in Inner Mongolia. In sampling area 1, a total of 2949 ticks from five species were collected, of which 75.8% (2234/2949) were *Ixodes persulcatus*, followed by *Haemaphysalis concinna* (15.0%, 441/2949), *Haemaphysalis douglasi* (4.5%, 134/2949), *Dermacentor nuttalli* (4.1%, 120/2949), and *Dermacentor silvarum* (0.7%, 20/2949). In sampling area 2, a total of 1334 ticks from three species were collected, of which 68.9% (919/1334) were *D*. *nuttalli*, followed by *Hyalomma asiaticum* (28.6%, 381/1334), and *Hyalomma marginatum* (5.7%, 76/1334). In sampling area 3, a total of 2173 ticks from four species were collected, of which 50.5% (1097/2173) were *D*. *nuttalli*, followed by *Hy*. *asiaticum* (35.3%, 766/2173), *Hy*. *marginatum* (12.3%, 268/2173), and *Rhipicephalus turanicus* (1.9%, 42/2173) (Table 2 and Fig 1).

**Table 2:** Tick Collection and co-infections.

Sample Area	Tick species	Tick collection (%)			Number of infections (%)			Number of co-infections (%)		
		Male	Female	All	Male	Female	All	Male	Female	All
Sample area 1	*Ha*. *concinna*	333 (21.3)	108 (7.8)	441 (15)	319 (95.6)	13 (12)	332 (75.3)	5 (1.6)	3 (23.1)	8 (2.4)
	*Ha*. *douglasi*	21 (1.3)	113 (8.2)	134 (4.5)	19 (90.5)	35 (31)	54 (40.3)	0	2 (5.7)	2 (3.7)
	*D*. *nuttalli*	85 (5.4)	35 (2.5)	120 (4.1)	75 (88.2)	34 (97.1)	109 (90.8)	0	0	0
	*D*. *silvarum*	1 (0.1)	19 (1.4)	20 (0.7)	1 (100)	11 (57.9)	12 (60)	0	0	0
	*I*. *persulcatus*	1125 (71.9)	1109 (80.1)	2234 (75.8)	735 (65.3)	679 (61.2)	1414 (63.3)	371 (50.5)	450 (66.3)	821 (58.1)
	Subtotal	1565 (53.1)	1384 (46.9)	2949	1149 (73.4)	772 (55.8)	1921 (65.1)	376 (32.7)	455 (58.9)	831 (43.3)
Sample area 2	*Hy*. *asiaticum*	217 (30.6)	164 (26.2)	381 (28.6)	104 (47.9)	119 (72.6)	223 (58.5)	0	11 (9.2)	11 (4.9)
	*Hy*. *marginatum*	33 (4.7)	43 (6.9)	76 (5.7)	13 (39.4)	27 (62.8)	40 (52.6)	0	0	0
	*D*. *nuttalli*	459 (64.7)	418 (66.9)	919 (68.9)	306 (66.7)	187 (44.7)	493 (53.6)	31 (10.1)	16 (8.6)	47 (9.5)
	Subtotal	709 (53.1)	625 (46.9)	1334	423 (59.7)	333 (53.3)	756 (56.7)	31 (7.2)	27 (8.1)	58 (7.6)
Sample area 3	*Hy*. *asiaticum*	490 (38.1)	276 (31.2)	766 (35.3)	223 (45.5)	106 (38.4)	329 (43)	0	0	0
	*Hy*. *marginatum*	96 (7.5)	172 (19.4)	268 (12.3)	90 (93.8)	159 (92.4)	249 (92.9)	0	0	0
	*D*. *nuttalli*	685 (53.2)	412 (46.5)	1097 (50.5)	411 (60)	293 (71.1)	704 (64.2)	38 (9.2)	35 (11.9)	73 (10.4)
	*R*. *turanicus*	16 (1.2)	26 (2.9)	42 (1.9)	4 (25.0)	3 (11.5)	7 (16.7)	0	0	0
	Subtotal	1287 (59.2)	886 (40.8)	2173	728 (56.6)	561 (63.3)	1289 (59.3)	38 (5.2)	35 (6.2)	73 (5.7)
	Total	3561 (55.2)	2895 (44.8)	6456	2300 (64.6)	1666 (57.6)	3966 (61.4)	445 (19.3)	517 (31)	962 (24.2)

### Prevalence of tick-borne pathogens in ticks

As shown in Table 2, tick-borne pathogens were detected in 61.4% (3966/6456) of all ticks collected from all sampling sites. The infection frequencies for male and female ticks were 64.6% (2300/3561) and 57.6% (1666/2895), respectively (Table 2). In sampling area 1, of the 2949 ticks collected, 1921 were infected, with an infection rate of 65.1% (1921/2949). The infection rates for different tick species varied greatly. *D*. *nuttalli* had the highest infection rate of 90.8% (109/120), followed by *Ha*. *concinna*, *I*. *persulcatus*, and *D*. *silvarum* with 75.3% (332/441), 63.3% (1414/2234), and 60.0% (12/20) infection rates, respectively. *Ha*. *douglasi* showed the lowest infection rate of 40.3% (54/134) compared with the other tick species (*χ*^2^ = 94.867, *P* < 0.001). In sampling area 2, of the 1334 ticks collected, 756 were PCR-positive, with an infection rate of 56.7% (756/1334). The infection rates of *Hy*. *asiaticum*, *D*. *nuttalli*, and *Hy*. *marginatum* were 58.5% (223/381), 53.6% (493/919), and 52.6% (40/76), respectively (*χ*^2^ = 2.770, *P* = 0.250). In sampling area 3, of the 2173 ticks collected, 1289 were PCR-positive, with an infection rate of 59.3% (1289/2173). The infection rate for different tick species varied greatly. *Hy*. *marginatum* had the highest infection rate of 92.9% (249/268), followed by *D*. *nuttalli* and *Hy*. *asiaticum* with infection rates of 64.2% (704/1097) and 43.0% (329/766), respectively. *R*. *turanicus* had the lowest infection rate of 16.7% (7/42) (*χ*^2^ = 252.747, *P* < 0.001) (Table 2 and Fig 1).

Sequencing of the PCR products revealed that the SFGR *gltA* gene shared 100% identity with *Rickettsia raoultii* (accession no. DQ365803), *Candidatus* R. tarasevichiae (accession no. MN450397), and *Rickettsia* sp. strain YN02 (accession no. KY411135). We performed *rOmpA* PCR of the *gltA* PCR-positive samples and confirmed that the results were consistent. Ehrlichial *p28*/*omp-1* multigenes were detected in *E*. *chaffeensis* (accession no. CP007480) (62.5%–100%), *E*. *ewingii* (accession no. AF287964) (65.5%–100%), *E*. *muris* (accession no. AB178807) (72.5%–100%), and *Ehrlichia* sp. strain HF565 (accession no. AB178803) (67.5%–100%). *Anaplasma p44*/*msp2* multigenes were detected in *Anaplasma phagocytophilum* (accession no. BAN28309) (67.5%–100%). The borrelial *flaB* and *glpQ* genes shared 100% identity with *Borrelia afzelii* strain HLJ01 (accession no. CP003882), *Borrelia garinii* strain NMJW1 (accession no. CP003866), *Borrelia* sp. HFW-21 (accession no. LC170020), and *B*. *miyamotoi* strain HT31 (accession no. AB900798). A recent study identified and reclassified *B*. *garinii* strain NMJW1 as *B*. *bavariensis* by whole genome sequencing and multilocus sequence typing (https://pubmlst.org/organisms/borrelia-spp). However, we did not perform either of these techniques in this study to further classify the *Borrelia* species detected from ticks. Thus, we temporally used the designation ‘*Borrelia* sp.’, which was identical to *B*. *garinii* strain NMJW1, to represent the *B*. *garinii*-complex in this study.

### Co-infections

Among the 6456 collected ticks, the overall prevalence of tick-borne pathogens was 61.4% (3966/6456), and the co-infection rate of the 3 regions was significantly different.

In sample area 1, of the 1921 infected ticks, co-infections were identified in 43.3% (831/1921). Among them, co-infection of *I*. *persulcatus* accounted for the majority of cases. In sample area 2, of the 756 infected ticks, co-infections were identified in 7.6% (58/756). Among them, co-infection of *D*. *nuttalli* and *Hy*. *asiaticum* accounted for 81.0% (47/58) and 19.0% (11/58) of cases, respectively. In sample area 3, of the 1289 infected ticks, all co-infections were detected with *D*. *nuttalli*, and the co-infection rate was 5.7% (73/1289). The co-infection rate in sample area 1 was significantly higher than that in sample areas 2 (*χ*^2^ = 317.145, *P* < 0.001) and 3 (*χ*^2^ = 530.261, *P* < 0.001) (Table 2).

### Pathogen detection and identification in co-infections

SFGR, *Ehrlichia*, *Anaplasma*, and *Borrelia* were detected in co-infections in all sampling sites. In sampling area 1, of the 831 co-infected ticks, most carried *C*. *R*. *tarasevichiae*, accounting for 86.2% (716/831), followed by the *B*. *garinii*-complex (including *B*. *bavariensis* and *B*. *garinii*), with a DNA-positive rate of 59.8% (497/831). In sampling area 2, of the 58 co-infected ticks, all carried the *B*. *garinii*-complex, followed by *R*. *raoultii*, at a rate of 72.4% (42/58). In sampling area 3, of the 73 co-infected ticks, all carried *R*. *raoultii*, followed by the *B*. *garinii*-complex, at a rate of 84.9% (62/73) (Table 3).

**Table 3:** Pathogen identified in co-infected Tick samples.

Bacterial species		Co-infected tick (%)		
		Sample area 1: 831 ticks	Sample area 2: 58 ticks	Sample area 3: 73 ticks
SFGR*^1^	*R*. *raoultii*	62 (7.5)	42 (72.4)	73 (100)
	*C*. *R*. *tarasevichiae*	716 (86.2)		
	*Rickettsia* sp. YN02		16 (27.6)	
*Ehrlichia*	*E*. *chaffeensis*	4 (0.5)		
	*E*. *ewingii*	27 (3.2)		
	*E*. *muris*	141 (17)	3 (8.3)	
	*Ehrlichia* sp. HF565	23 (2.7)		
*Anaplasma*	*A*. *phagocytophilum*	231 (27.1)		
*Borrelia*	*B*. *afzelii*	2 (0.2)		10 (13.7)
	*B*. *miyamotoi*	179 (21.5)		1 (1.4)
	*B*. *garinii-*complex	497 (59.8)	58 (100)	62 (84.9)
	*Borrelia* sp. HFW-21	7 (0.8)		

*1: SFGR (Spotted fever group rickettsiae).

### Co-infection between different pathogen types

In sampling area 1, co-infection between *C*. *R*. *tarasevichiae* and the *B*. *garinii*-complex was the most common, with a co-infection rate of 4.6% (297/6456), followed by *C*. *R*. *tarasevichiae* and *A*. *phagocytophilum* (1.7%; 108/6456). In sample areas 2 and 3, co-infection was most frequent for *R*. *raoultii* and the *B*. *garinii*-complex (39/6456; 0.6% and 63/6456; 1.0%, respectively) (Table 4).

**Table 4:** Pathogen co-infection type in Tick samples.

Pathogen associations	Sample area 1			Sample area 2			Sample area 3			Total No.		
	M*^1^	F*^2^	Total	M	F	Total	M	F	Total	M	F	Total
*R*. *raooltii*+*E*. *muris*	3		3							3		3
*R*. *raooltii*+*A*. *phagocytophilum*	7	1	8							7	1	8
*R*. *raooltii*+*B*. *garinii-*complex	10	20	30	27	12	39	28	35	63	65	67	132
*R*. *raooltii*+*B*. *miyamotoi*	1		1							1		1
*R*. *raooltii*+*B*. *afzelii*							10	0	10	10		10
*Rickettsia sp*.*+B*. *garinii-*complex				2	14	16				2	14	16
*C*. *R*. *tarasevichiae+E*. *muris*	29	21	50							29	21	50
*C*. *R*. *tarasevichiae*+*E*. *chaffeensis*	1	3	4							1	3	4
*C*. *R*. *tarasevichiae*+*E*. *ewingii*	1	2	3							1	2	3
*C*. *R*. *tarasevichiae*+*A*. *phagocytophilum*	62	46	108							62	46	108
*C*. *R*. *tarasevichiae*+*B*. *garinii-*complex	107	190	297							119	178	297
*C*. *R*. *tarasevichiae*+*B*. *miyamotoi*	35	29	64							35	29	64
*E*. *muris+A*. *phagocytophilum*	3	3	6							3	3	6
*E*. *muris+B*. *garinii-*complex	1	1	2							1	1	2
*E*. *muris+B*. *miyamotoi*	4	3	7							4	3	7
*E*. *ewingii+B*. *garinii-*complex	7		7							7		7
*A*. *phagocytophilum+B*. *garinii-*complex	3	3	6							3	3	6
*A*. *phagocytophilum+B*. *miyamotoi*	2	3	5							2	3	5
*B*. *garinii-*complex*+B*. *miyamotoi*	3	2	5							3	2	5
*B*. *miyamotoi+B*. *afzelii*	0	2	2							0	2	2
*B*. *miyamotoi+Borrelia* sp.	3	4	7							3	4	7
*R*. *raooltii+E*. *muris+A*. *phagocytophilum*	1		1							1		1
*R*. *raooltii+E*. *muris+B*. *garinii-*complex	2	4	6	2	1	3				4	5	9
*R*. *raooltii+A*. *phagocytophilum+B*. *miyamotoi*	1	8	9							1	8	9
*R*. *raooltii+B*. *garinii-*complex*+B*. *miyamotoi*	1	2	3							1	2	3
*C*. *R*. *tarasevichiae+E*. *muris+A*. *phagocytophilum*	9	8	17							9	8	17
*C*. *R*. *tarasevichiae*+*E*. *muris+B*. *garinii-*complex	15	15	30							15	15	30
*C*. *R*. *tarasevichiae*+*E*. *muris+B*. *miyamotoi*	3	1	4							3	1	4
*C*. *R*. *tarasevichiae*+*E*. *ewingii+A*. *phagocytophilum*	1		1							1		1
*C*. *R*. *tarasevichiae*+*E*. *ewingii+B*. *garinii-*complex	4	7	11							4	7	11
*C*. *R*. *tarasevichiae*+*Ehrlichia sp*.*+B*. *garinii-*complex	6	7	13							6	7	13
*C*. *R*. *tarasevichiae*+*Ehrlichia sp*.*+B*. *miyamotoi*	4	5	9							4	5	9
*C*. *R*. *tarasevichiae*+*A*. *phagocytophilum+B*. *garinii-*complex	8	17	25							8	17	25
*C*. *R*. *tarasevichiae*+*A*. *phagocytophilum+B*. *miyamotoi*	6	13	19							6	13	19
*C*. *R*. *tarasevichiae+B*. *garinii-*complex*+B*. *miyamotoi*	14	22	36							14	22	36
*E*. *ewingii+A*. *phagocytophilum+B*. *garinii-*complex		2	2							0	2	2
*E*. *ewingii+B*. *garinii-*complex*+B*. *miyamotoi*	2		2							2		2
*A*. *phagocytophilum+B*. *garinii-*complex*+B*. *miyamotoi*	1	1	2							1	1	2
*R*. *raooltii+E*. *muris+A*. *phagocytophilum+B*. *miyamotoi*		1	1							0	1	1
*C*. *R*. *tarasevichiae+E*. *muris*+*A*. *phagocytophilum+B*. *garinii-*complex	1		1							1		1
*C*. *R*. *tarasevichiae+E*. *muris*+*A*. *phagocytophilum+B*. *miyamotoi*	3	1	4							3	1	4
*C*. *R*. *tarasevichiae+E*. *muris*+*B*. *garinii-*complex*+B*. *miyamotoi*	5	3	8							5	3	8
*C*. *R*. *tarasevichiae*+*A*. *phagocytophilum+B*. *garinii-*complex*+B*. *miyamotoi*	4	4	8							4	4	8
*C*. *R*. *tarasevichiae*+*E*. *ewingii+A*. *phagocytophilum+B*. *garinii-*complex	1	1	2							1	1	2
*C*. *R*. *tarasevichiae*+*Ehrlichia* sp.+*A*. *phagocytophilum+B*. *miyamotoi*	1		1							1	0	1
*C*. *R*. *tarasevichiae*+*E*. *muris*+*A*. *phagocytophilum*+*B*. *garinii-*complex*+B*. *miyamotoi*	1		1							1	0	1
Total	376	455	831	31	27	58	38	35	73	445	517	962

*1: Male ticks, *2: Female ticks.

In sampling area 1, triple pathogen co-infection was identified in 190 ticks, 36 of which were infected with *C*. *R*. *tarasevichiae*, the *B*. *garinii*-complex, and *B*. *miyamotoi* (36/6456; 0.6%), followed by *C*. *R*. *tarasevichiae*, *E*. *muris*, and the *B*. *garinii*-complex (30/6456; 0.5%). In sampling area 2, only three ticks showed triple pathogen co-infection, comprising *R*. *raoultii*, *E*. *muris*, and the *B*. *garinii*-complex. In sampling area 3, no ticks were found to be co-infected with three different pathogens (Table 4).

In sampling area 1, 25 ticks were detected to be co-infected with four different pathogens, eight of which were infected with *C*. *R*. *tarasevichiae*, *E*. *muris*, the *B*. *garinii*-complex, and *B*. *miyamotoi*, and eight were infected with *C*. *R*. *tarasevichiae*, *A*. *phagocytophilum*, the *B*. *garinii*-complex, and *B*. *miyamotoi*. In sampling areas 2 and 3, no ticks were found to be co-infected with four different pathogens (Table 4).

Only in sampling area 1, was one tick found to be co-infected with five different pathogens, namely *C*. *R*. *tarasevichiae*, *E*. *muris*, *A*. *phagocytophilum*, the *B*. *garinii*-complex, and *B*. *miyamotoi* (Table 4).

## Discussion

Co-infection with tick-borne pathogens has been suggested to reflect the fact that ticks can carry and transmit multiple pathogens and the need for ticks to switch to different hosts to complete their entire growth process, thereby increasing the likelihood of acquiring different pathogens from different hosts [23,24]. Co-infection can occur when a livestock tick carrying multiple pathogens or multiple ticks carrying multiple pathogens bite a person in succession [25].

In this study, a comprehensive investigation of the epidemiological status of 12 tick-borne bacterial pathogens of four genera was performed in co-infected ticks isolated in northern China. The important findings of our study were as follows. (1) The identification of a variety of tick-borne bacterial pathogens, with an overall high prevalence rate (61.4% of ticks infected). (2) The frequency of co-infection. Among infected ticks, 24.2% were co-infected, with co-infection of *C*. *R*. *tarasevichiae* and the *B*. *garinii*-complex being the most common. (3) The unexpected high infection and co-infection rates of ticks collected from the forest region of eastern Inner Mongolia (sample area 1). (4) The significant changes in the ecological and geographical distribution of the main dominant tick species, and the corresponding increase in pathogen diversity between the Gobi Desert and the semi-desertification steppe, and the grasslands and forest. Together, these results indicate the significant potential threat to public health of tick-borne pathogens.

In this study, we detected the *B*. *garinii*-complex in ticks. In a previous study, *B*. *garinii*, comprising of the formerly designated *B*. *garinii* and *B*. *bavariensis*, was reported to be distributed across China [26]. Although these two *Borrelia* spp. are distinguishable by multi-locus sequence typing [27], they cannot be distinguished by *flaB*-sequencing because of its low resolution. Therefore, it is highly probable that both the former *B*. *garinii* and *B*. *bavariensis* have previously been designated as *B*. *garinii*.

Inner Mongolia covers a wide geographic area from east to west, and the ecological environments across this region are therefore quite distinct. The vegetation that constitutes the tick habitat changes from east to west, from forests to grasslands to semi-desert grasslands to deserts. The distribution of tick species is closely related to host species and the ecological environment and, consequently, the risk of human tick-borne infection varies from region to region [4–6]. *I*. *persulcatus* was the dominant tick species in the eastern forest region (sample area 1). *I*. *persulcatus* is a typical forest tick species, which is dominant in conifer and broadleaved mixed forest, and its host range is wide, including domestic or wild medium and small mammals. The distribution of *I*. *persulcatus* covers Inner Mongolia [13,14], mainland China [28], and more specifically the southwest and northeast of China [29–32], among other places. *D*. *nuttalli* was the dominant tick species in the central and western grasslands (sample areas 2 and 3). *D*. *nuttalli* inhabits the arid semi-desert steppe regions, mainly parasitizing livestock and humans. The distribution of *D*. *nuttalli* covers Inner Mongolia [12,14], Gansu [33], and the southwest and northeast of China [29,31,32], among other places.

In sample area 1, *C*. *R*. *tarasevichiae* was found to reside with other pathogens in 23 co-infection patterns, accounting for 81.7% of co-infections. Among them, the co-infection of *C*. *R*. *tarasevichiae* and the *B*. *garinii*-complex was most common, followed by *C*. *R*. *tarasevichiae* and *A*. *phagocytophilum*. *C*. *R*. *tarasevichiae* was first identified in *I*. *persulcatus* ticks from various sites in Russia in 2001 [34]. Human infection with *C*. *R*. *tarasevichiae* was first reported in northeastern China in 2012 [35] and is widely distributed in eastern and northeastern China [11,36,37]. The forest region in northeast China is close to Russia in terms of geography. With the development of tourism, animal husbandry, and logging, human activities have increased the opportunity for humans, livestock, and ticks to come into contact, possibly creating conditions for the spread of *C*. *R*. *tarasevichiae*. A previous study found that *B*. *burgdorferi* sensu lato infection in ticks and mice in the Greater Khingan Mountains forest region of Inner Mongolia is mainly caused by *B*. *garinii* [38]. Pan and colleagues [39] also found that the co-infection rate of *C*. *R*. *tarasevichiae* and *B*. *burgdorferi* sensu lato was high (20%) in *I*. *persulcatus* in Heilongjiang Province. Co-infection with *C*. *R*. *tarasevichiae* has been reported to aggravate disease symptoms and has been linked with mortality [11]. Our results suggest that *C*. *R*. *tarasevichiae* has a high rate of co-infection with other pathogens, which highlights the importance of considering *C*. *R*. *tarasevichiae* in the differential diagnosis of other tick-borne pathogens in endemic regions. In sample areas 2 and 3, the most frequent genospecies association was between *R*. *raoultii* and *B*. *garinii*. Co-infection with *B*. *garinii* is relatively common [1,40]. *R*. *raoultii* is widely distributed in the steppe of central and western Inner Mongolia, and *D*. *nuttalli* is the main vector and host. In central and western Inner Mongolia, the grassland is arid and the vegetation coverage rate is low, but the parasitism rate of *D*. *nuttalli* remains high in spring and summer, which seriously affects local livestock production. The infection and co-infection rates in ticks from sample area 1 (deciduous and mixed forest vegetation) were the most serious, thus highlighting a significant disease risk in this area, a heavily frequented recreational area and tourist hotspot. In addition, the detection rate of *Rickettsia* and *Borrelia* was high in ticks in this study, indicating an increased probability of their co-infection with other pathogens.

In this study, the predominant host among co-infection cases was *I*. *persulcatus*, accounting for more than 85% of co-infections. It has been confirmed that *I*. *persulcatus* can be naturally infected with a variety of pathogens. Fu and colleagues [41] found that at least 40% of *I*. *persulcatus* individuals were co-infected, including both double and triple infections.

Co-infections might have consequences in terms of pathogen co-transmission [24], and the high co-infection rate among ticks poses a health threat to humans and livestock [3,42]. The clinical presentation of tick-borne-associated bacterial infections is similar, therefore, diagnosis is challenging and co-infection can be easily missed. The co-infecting pathogens might play different roles within their respective host, thus modulating disease severity [43,44].

Ecological changes and social development may have contributed to the emergence of the tick-borne diseases by placing people in increasing contact with ticks and potential animal reservoirs. Therefore, medical personnel should be trained in identified tick-borne disease hotspots (sample area 1), to improve the detection and identification of TBRD and treatment strategies to reduce the fatality rate linked to co-infection. Disease control and prevention personnel should also be trained to conduct epidemiological investigations and to control the spread and prevalence of outbreaks.

Our findings highlight the severity of tick-borne pathogen infections in the eastern forest region through the collection of field data across all regions of Inner Mongolia from 2015 to 2019. In response, it is hoped that relevant departments can pay increased attention to the co-infection of tick-borne pathogens, and conduct timely screening and clinical treatment for common co-infection patterns to avoid the occurrence of more serious complications.

In this study, sequencing was performed on some samples and *R*. *raoultii* was detected from *D*. *nuttalli* and *C*. *R*. *tarasevichiae* from *I*. *persulcatus*. Data from neighboring countries showed the presence of *Rickettsia helvetica* [45], but we failed to detect it within the scope of this investigation. Therefore, the genotypes of rickettsiae may be incomplete, and we will continue to expand the sample size to be sequenced for verification.

## References

[1] Raileanu C, Moutailler S, Pavel I, Porea D, Mihalca AD, Savuta G, Vayssier-Taussat M. *Borrelia* Diversity and Co-infection with Other Tick Borne Pathogens in Ticks. *Front Cell Infect Microbiol*. 2017;7:36. doi: 10.3389/fcimb.2017.00036 28261565 PMC5306127

[2] Blazejak K, Raulf MK, Janecek E, Jordan D, Fingerle V, Strube C. Shifts in *Borrelia burgdorferi* (s.l.) geno-species infections in *Ixodes ricinus* over a 10-year surveillance period in the city of Hanover (Germany) and *Borrelia miyamotoi*-specific Reverse Line Blot detection. *Parasit Vectors*. 2018;11(1):304. doi: 10.1186/s13071-018-2882-9 29776377 PMC5960134

[3] Chen X, Li F, Yin Q, Liu W, Fu S, He Y, Lei W, Xu S, Liang G, Wang S, Yang G, Qi X, Wang H. Epidemiology of tick-borne encephalitis in China, 2007–2018. *PLoS One*. 2019;14(12):e0226712. doi: 10.1371/journal.pone.0226712 31877145 PMC6932775

[4] Li Y, Wang J, Gao M, Fang L, Liu C, Lyu X, Bai Y, Zhao Q, Li H, Yu H, Cao W, Feng L, Wang Y, Zhang B. Geographical Environment Factors and Risk Assessment of Tick-Borne Encephalitis in Hulunbuir, Northeastern China. *Int J Environ Res Public Health*. 2017;14(6):569. doi: 10.3390/ijerph14060569 28587151 PMC5486255

[5] Fang LQ, Liu K, Li XL, Liang S, Yang Y, Yao HW, Sun RX, Sun Y, Chen WJ, Zuo SQ, Ma MJ, Li H, Jiang JF, Liu W, Yang XF, Gray GC, Krause PJ, Cao WC. Emerging tick-borne infections in mainland China: an increasing public health threat. *Lancet Infect Dis*. 2015;15(12):1467–1479. doi: 10.1016/S1473-3099(15)00177-2 26453241 PMC4870934

[6] Kilpatrick AM, Randolph SE. Drivers, dynamics, and control of emerging vector-borne zoonotic diseases. *Lancet*. 2012;380(9857):1946–55. doi: 10.1016/S0140-6736(12)61151-9 23200503 PMC3739480

[7] Mitchell PD, Reed KD, Hofkes JM. Immunoserologic evidence of coinfection with *Borrelia burgdorferi*, *Babesia microti*, and human granulocytic *Ehrlichia* species in residents of Wisconsin and Minnesota. *J Clin Microbiol*. 1996;34(3):724–7. doi: 10.1128/jcm.34.3.724–727.1996 8904446 PMC228878

[8] Süss J, Schrader C, Abel U, Bormane A, Duks A, Kalnina V. Characterization of tick-borne encephalitis (TBE) foci in Germany and Latvia (1997–2000). *Int J Med Microbiol*. 2002;291 Suppl 33:34–42. doi: 10.1016/s1438-4221(02)80007-8 12141755

[9] Dibernardo A, Cote T, Ogden NH, Lindsay LR. The prevalence of *Borrelia miyamotoi* infection, and co-infections with other *Borrelia* spp. in *Ixodes scapularis* ticks collected in Canada. *Parasit Vectors*. 2014;7:183. doi: 10.1186/1756-3305-7-183 24731287 PMC4001108

[10] Lu QB, Li H, Zhang PH, Cui N, Yang ZD, Fan YD, Cui XM, Hu JG, Guo CT, Zhang XA, Liu W, Cao WC. Severe Fever with Thrombocytopenia Syndrome Complicated by Co-infection with Spotted Fever Group Rickettsiae, China. *Emerg Infect Dis*. 2016;22(11):1957–1960. doi: 10.3201/eid2211.161021 27767921 PMC5088031

[11] Liu W, Li H, Lu QB, Cui N, Yang ZD, Hu JG, Fan YD, Guo CT, Li XK, Wang YW, Liu K, Zhang XA, Yuan L, Zhao PY, Qin SL, Cao WC. *Candidatus* Rickettsia tarasevichiae Infection in Eastern Central China: A Case Series. *Ann Intern Med*. 2016;164(10):641–8. doi: 10.7326/M15-2572 27019406

[12] Gaowa Wulantuya, Yin X, Cao M, Guo S, Ding C, Lu Y, Luo J, Kawabata H, Ando S, Su H, Shimada M, Takamoto N, Shimamura Y, Masuda S, Ohashi N. Case of Human Infection with *Anaplasma phagocytophilum* in Inner Mongolia, China. *Jpn J Infect Dis*. 2018;71(2):155–157. doi: 10.7883/yoken.JJID.2017.450 29491236

[13] Gaowa Wulantuya, Sato K, Liu D, Cui Y, Yin X, Zhang L, Li H, Wang T, Liu R, Wu L, Lu S, Gao T, Zhang Z, Cao M, Wang G, Li C, Yan D, Ohashi N, Ando S, Kawabata H. Surveillance of *Borrelia miyamotoi*-carrying ticks and genomic analysis of isolates in Inner Mongolia, China. *Parasit Vectors*. 2021;14(1):368. doi: 10.1186/s13071-021-04809-z 34274015 PMC8285808

[14] Gao Y, Lv XL, Han SZ, Wang W, Liu Q, Song M. First detection of *Borrelia miyamotoi* infections in ticks and humans from the northeast of Inner Mongolia, China. *Acta Trop*. 2021;217:105857. doi: 10.1016/j.actatropica.2021.105857 33582142

[15] Razanske I, Rosef O, Radzijevskaja J, Bratchikov M, Griciuviene L, Paulauskas A. Prevalence and co-infection with tick-borne *Anaplasma phagocytophilum* and *Babesia* spp. in red deer (*Cervus elaphus*) and roe deer (*Capreolus capreolus*) in Southern Norway. *Int J Parasitol Parasites Wildl*. 2019;8:127–134. doi: 10.1016/j.ijppaw.2019.01.003 30766793 PMC6360459

[16] Bursakov SA, Kovalchuk SN. Co-infection with tick-borne disease agents in cattle in Russia. *Ticks Tick Borne Dis*. 2019;10(3):709–713. doi: 10.1016/j.ttbdis.2019.03.004 30878569

[17] Takano A, Fujita H, Kawabata H, et al. Construction of a DNA database for ticks collected in Japan: application of molecular identification based on the mitochondrial 16S rDNA gene. *Medical Entomology and Zoology*. 2014;65(1):13–21. doi: 10.7601/mez.65.13

[18] Gaowa, Ohashi N, Aochi M, Wuritu D, Wu, Yoshikawa Y, Kawamori F, Honda T, Fujita H, Takada N, Oikawa Y, Kawabata H, Ando S, Kishimoto T. *Rickettsiae* in ticks, Japan, 2007–2011. *Emerg Infect Dis*. 2013;19(2):338–40. doi: 10.3201/eid1902.120856 23460996 PMC3559048

[19] Gaowa, Yin X, Guo S, Ding C, Cao M, Kawabata H, Sato K, Ando S, Fujita H, Kawamori F, Su H, Shimada M, Shimamura Y, Masuda S, Ohashi N. Spotted Fever Group Rickettsiae in Inner Mongolia, China, 2015–2016. *Emerg Infect Dis*. 2018;24(11):2105–2107. doi: 10.3201/eid2411.162094 30334715 PMC6200000

[20] Barbour AG, Bunikis J, Travinsky B, Hoen AG, Diuk-Wasser MA, Fish D, Tsao JI. Niche partitioning of *Borrelia burgdorferi* and *Borrelia miyamotoi* in the same tick vector and mammalian reservoir species. *Am J Trop Med Hyg*. 2009;81(6):1120–31. doi: 10.4269/ajtmh.2009.09–0208 19996447 PMC2841027

[21] Takano A, Toyomane K, Konnai S, Ohashi K, Nakao M, Ito T, Andoh M, Maeda K, Watarai M, Sato K, Kawabata H. Tick Surveillance for Relapsing Fever Spirochete *Borrelia miyamotoi* in Hokkaido, Japan. *PLoS One*. 2014;9(8): e104532. 25111141 10.1371/journal.pone.0104532 PMC4128717

[22] Kumar S, Stecher G, Tamura K. MEGA7: Molecular evolutionary geneticsanalysis version 7.0 for bigger datasets. *Mol Biol Evol*. 2016;33(7):1870–4. doi: 10.1093/molbev/msw054 27004904 PMC8210823

[23] Chen Z, Liu Q, Liu JQ, Xu BL, Lv S, Xia S, Zhou XN. Tick-borne pathogens and associated co-infections in ticks collected from domestic animals in central China. *Parasit Vectors*. 2014;7:237. doi: 10.1186/1756-3305-7-237 24886497 PMC4045914

[24] Pettersson J. The Origin of the Genus Flavivirus and the Ecology of Tick-Borne Pathogens. *Biological Sciences*. 2013. ISBN 978-91-554-8814-7.

[25] Moutailler S, Valiente Moro C, Vaumourin E, Michelet L, Tran FH, Devillers E, Cosson JF, Gasqui P, Van VT, Mavingui P, Vourc’h G, Vayssier-Taussat M. Co-infection of Ticks: The Rule Rather Than the Exception. *PLoS Negl Trop Dis*. 2016;10(3):e0004539. doi: 10.1371/journal.pntd.0004539 26986203 PMC4795628

[26] Takano A, Nakao M, Masuzawa T, Takada N, Yano Y, Ishiguro F, Fujita H, Ito T, Ma X, Oikawa Y, Kawamori F, Kumagai K, Mikami T, Hanaoka N, Ando S, Honda N, Taylor K, Tsubota T, Konnai S, Watanabe H, Ohnishi M, Kawabata H. Multilocus sequence typing implicates rodents as the main reservoir host of human-pathogenic *Borrelia garinii* in Japan. *J Clin Microbiol*. 2011;49(5):2035–9. doi: 10.1128/JCM.02544-10 21411595 PMC3122701

[27] Margos G, Wilske B, Sing A, Hizo-Teufel C, Cao WC, Chu C, Scholz H, Straubinger RK, Fingerle V. *Borrelia bavariensis* sp. nov. is widely distributed in Europe and Asia. *Int J Syst Evol Microbiol*. 2013;63(11):4284–4288. doi: 10.1099/ijs.0.052001–0 23838444

[28] Sun RX, Lai SJ, Yang Y, Li XL, Liu K, Yao HW, Zhou H, Li Y, Wang LP, Mu D, Yin WW, Fang LQ, Yu HJ, Cao WC. Mapping the distribution of tick-borne encephalitis in mainland China. *Ticks Tick Borne Dis*. 2017;8(4):631–639. doi: 10.1016/j.ttbdis.2017.04.009 28461151

[29] Liu H, Liang X, Wang H, Sun X, Bai X, Hu B, Shi N, Wang N, Zhang X, Huang L, Liao J, Huang F, Zhang H, Si X, Huang S, Jin N, Liu Q, Li L. Molecular evidence of the spotted fever group Rickettsiae in ticks from Yunnan Province, *Southwest China*. *Exp Appl Acarol*. 2020;80(3):339–348. doi: 10.1007/s10493-020-00467-5 31925589

[30] Shao JW, Zhang XL, Li WJ, Huang HL, Yan J. Distribution and molecular characterization of rickettsiae in ticks in Harbin area of Northeastern China. *PLoS Negl Trop Dis*. 2020;14(6):e0008342. doi: 10.1371/journal.pntd.0008342 32497120 PMC7272007

[31] Zhang XC, Yang ZN, Lu B, Ma XF, Zhang CX, Xu HJ. The composition and transmission of microbiome in hard tick, *Ixodes persulcatus*, during blood meal. *Ticks Tick Borne Dis*. 2014;5(6):864–70. doi: 10.1016/j.ttbdis.2014.07.009 25150725

[32] Wei F, Song M, Liu H, Wang B, Wang S, Wang Z, Ma H, Li Z, Zeng Z, Qian J, Liu Q. Molecular Detection and Characterization of Zoonotic and Veterinary Pathogens in Ticks from Northeastern China. *Front Microbiol*. 2016;7:1913. doi: 10.3389/fmicb.2016.01913 27965644 PMC5126052

[33] Liu Z, Shi S, Wang D, Yang Y, Luo Y. Infection rates of *Borrelia burgdorferi* in ticks in Gansu, China. *Systematic and Applied Acarology*. 1997;2:241. doi: 10.11158/15684

[34] Shpynov S, Fournier PE, Rudakov N, Raoult D. "*Candidatus* Rickettsia tarasevichiae" in *Ixodes persulcatus* ticks collected in Russia. *Ann N Y Acad Sci*. 2003;990:162–72. doi: 10.1111/j.1749-6632 12860621

[35] Jia N, Zheng YC, Jiang JF, Ma L, Cao WC. Human infection with *Candidatus* Rickettsia tarasevichiae. *N Engl J Med*. 2013;369(12):1178–80. doi: 10.1056/NEJMc1303004 24047080

[36] Yi S, Hongrong J, Wuchun C, Weiming F, Wendong J, Xin W. Prevalence of *Candidatus* Rickettsia tarasevichiae-Like Bacteria in Ixodid Ticks at 13 Sites on the Chinese-Russian Border. *J Med Entomol*. 2014;51(6):1304–7. doi: 10.1603/ME13189 26309321

[37] Yuan TT, Ma L, Jiang BG, Fu WM, Sun Y, Jia N, Jiang JF. First Confirmed Infection of *Candidatus* Rickettsia Tarasevichiae in Rodents Collected from Northeastern China. *Vector Borne Zoonotic Dis*. 2020;20(2):88–92. doi: 10.1089/vbz.2019.2443 31453762

[38] Chu CY, He J, Wang JB, Hasen GW, Zhang PH, Wu XM, Zhao QM, Jiang BG, Gao Y, Cao WC. Investigation on *Borrelia burgdorferi* sensu lato in ticks and rodents collected in Da Xing-An Mountains Forest areas of China. Zhonghua Liu Xing Bing Xue Za Zhi. 2006;27(8):681–4. . 17172108

[39] Pan YP, Yan JF, Niu QL, Mirza OA, Zhai BT, Zeng QY, Hong Y. Study on *Borrelia burgdorferi* sensu lato and spotted fever group *Rickettsia* in *Ixodes persulcatus* in Heilongjiang Province. *Chinese Veterinary Science*, 2017;47(1):31–37.doi: CNKI:SUN:ZGSY.0.2017-01-005.

[40] Jiao J, Lu Z, Yu Y, Ou Y, Fu M, Zhao Y, Wu N, Zhao M, Liu Y, Sun Y, Wen B, Zhou D, Yuan Q, Xiong X. Identification of tick-borne pathogens by metagenomic next-generation sequencing in *Dermacentor nuttalli* and *Ixodes persulcatus* in Inner Mongolia, China. *Parasit Vectors*. 2021;14(1):287. doi: 10.1186/s13071-021-04740-3 34044867 PMC8161991

[41] Fu FX, Guo W, Zhang Y, et al. Advances in epidemiological reseach on tick borme infectious diseases. *International Journal of Epidemiology and Infectious Diseases*. 2012;39(4):285–288. doi: 10.3760/cma.j.issn.1673-4149.2012.04.020

[42] Baneth G. Tick-borne infections of animals and humans: a common ground. *Int J Parasitol*. 2014;44(9):591–6. doi: 10.1016/j.ijpara.2014.03.011 24846527

[43] Diuk-Wasser MA, Vannier E, Krause PJ. Coinfection by *Ixodes* Tick-Borne Pathogens: Ecological, Epidemiological, and Clinical Consequences. *Trends Parasitol*. 2016;32(1):30–42. doi: 10.1016/j.pt.2015.09.008 26613664 PMC4713283

[44] States SL, Huang CI, Davis S, Tufts DM, Diuk-Wasser MA. Co-feeding transmission facilitates strain coexistence in *Borrelia burgdorferi*, the Lyme disease agent. *Epidemics*. 2017;19:33–42. doi: 10.1016/j.epidem.2016.12.002 28089780 PMC5474356

[45] Kartashov MY, Glushkova LI, Mikryukova TP, Korabelnikov IV, Egorova YI, Tupota NL, Protopopova EV, Konovalova SN, Ternovoi VA, Loktev VB. Detection of *Rickettsia helvetica* and *Candidatus* R. tarasevichiae DNA in *Ixodes persulcatus* ticks collected in Northeastern European Russia (Komi Republic). *Ticks Tick Borne Dis*. 2017;8(4):588–592. doi: 10.1016/j.ttbdis.2017.04.001 28433730

