# Supplementary material for: Co-infection of tick-borne bacterial pathogens in ticks in Inner Mongolia, China
Source: PLoS Negl Trop Dis. 2023 Mar 9;17(3):e0011121. doi: 10.1371/journal.pntd.0011121 (PMC10030021; doi:10.1371/journal.pntd.0011121)
Supplement: Attachment — Submitted filename: Response to Reviewers.docx [file pntd.0011121.s001.docx]

Dear editor，

We really appreciate your great effort in reviewing our manuscript and provide us an opportunity to improve the quality of our paper.

According to the editor/reviewers suggestions, we have carefully revised and improved our manuscript. All authors declared that they have no competing interests.

Your comments and suggestions really helped us a lot. We have put great efforts on them.If have anyinformation I can provide, please don’t hesitate to contact me.

Thank you again for your time and patience. Looking forward to hearing from you.

Sincerely yours,

Dan Liu,

Gaowa.

Manuscript ID:PNTD-D-22-00256

Type of manuscript: Article

Title: Tick-borne bacterial pathogens in ticks collected in Inner Mongolia, China

**Reviewer reports:**

**Reviewer's Responses to Questions**

**Key Review Criteria Required for Acceptance?**

**As you describe the new analyses required for acceptance, please consider the following:**

**Methods**

**-Are the objectives of the study clearly articulated with a clear testable hypothesis stated?**

**-Is the study design appropriate to address the stated objectives?**

**-Is the population clearly described and appropriate for the hypothesis being tested?**

**-Is the sample size sufficient to ensure adequate power to address the hypothesis being tested?**

**-Were correct statistical analysis used to support conclusions?**

**-Are there concerns about ethical or regulatory requirements being met?**

Reviewer #1: Multivariate analysis would be better to add for analysis of co-infection.

**Response:** In response to the reviewer’s comment, we tried to use statistical models to include some potential influencing factors for multivariate analysis. However, based on the incomplete data collected in the early stage, the difficulty of multivariate analysis is slightly greater, which will be gradually improved in the subsequent research.

In this study was to describe the regional differences in co-infection outcomes, and found that pathogen diversity varied with ecogeographic differences such as gobi desert, grassland and forest. In addition, some samples were collected on timescales longer and involved a wide range, and some collection sites did not record other detailed data except ecological characteristics, there are certain difficulties in multivariate analysis at present. In future studies, we will regression tick collection sites, record detailed variable factors that may affect tick distribution and infection, and then analyze them.

Reviewer #2: -Please verify that only verbal consent is needed/required from the animal owners (no written consent).

-Please state the types of livestock involved.

-Please elaborate on the "animal physical examination method".

-Please include the taxonomic keys used for tick species identification.

-Please clarify the reference(s) for PCR protocol/gene used in the study and cite accordingly.

-Re-check whether the procedure for sequence analysis has been described adequately/thoroughly.

**Response:**

**(1)** The tick samples we collected were all confirmed by the animal owners.

**(2)** The livestock are cattle、goat、sheep、camel and horse.

**(3)** From March to May every year, tick is the most active season in Inner Mongolia grassland, and a large number of livestock are parasitized by ticks. We catch the animals with the cooperation of livestock owners, and the tick that live on their bodies are removed with tweezers.

**(4)**The tick species, sex, and stage were determined using a microscope, according to morphological keys.

**(5)** We have added reference for PCR protocol/gene used in the study.

Ref 12,17,18,19,20 and 21.

[Ref] Gaowa, Wulantuya, Yin X, Cao M, Guo S, Ding C, Lu Y, Luo J, Kawabata H, Ando S, Su H, Shimada M, Takamoto N, Shimamura Y, Masuda S, Ohashi N. Case of Human Infection with *Anaplasma phagocytophilum* in Inner Mongolia, China. Jpn J Infect Dis. 2018;71(2):155-157. doi: 10.7883/yoken.JJID.2017.450.

[Ref] Barbour AG, Bunikis J, Travinsky B, Hoen AG, Diuk-Wasser MA, Fish D, Tsao JI. Niche partitioning of Borrelia burgdorferi and *Borrelia miyamotoi* in the same tick vector and mammalian reservoir species. Am J Trop Med Hyg. 2009;81(6):1120-31. doi: 10.4269/ajtmh.2009.09-0208.

[Ref] Gaowa, Yin X, Guo S, Ding C, Cao M, Kawabata H, Sato K, Ando S, Fujita H, Kawamori F, Su H, Shimada M, Shimamura Y, Masuda S, Ohashi N. Spotted Fever Group *Rickettsiae* in Inner Mongolia, China, 2015-2016. Emerg Infect Dis. 2018;24(11):2105-2107. doi: 10.3201/eid2411.162094.

[Ref] Gaowa, Ohashi N, Aochi M, Wuritu D, Wu, Yoshikawa Y, Kawamori F, Honda T, Fujita H, Takada N, Oikawa Y, Kawabata H, Ando S, Kishimoto T. *Rickettsiae* in ticks, Japan, 2007–2011. Emerg Infect Dis. 2013;19(2):338-40. doi: 10.3201/eid1902.120856.

[Ref] Takano A, Fujita H, Kawabata H, et al. Construction of a DNA database for ticks collected in Japan: application of molecular identification based on the mitochondrial 16S rDNA gene. Medical Entomology and Zoology. 2014;65(1):13-21.doi: 10.7601/mez.65.13.

[Ref] Takano A, Toyomane K, Konnai S, OhashiK, Nakao M, Ito T, Andoh M, Maeda K, Watarai M, Sato K, Kawabata H. Tick Surveillance for Relapsing Fever Spirochete Borrelia miyamotoi in Hokkaido, Japan. PLoS One. 2014;9(8): e104532.

**(6)** About the re-check whether the procedure for sequence analysis has been described adequately, in the method"PCR Products purification and Sequence Analysis", we had added the content and reference related to the procedure for sequence analysis on the basis of the original content.

Reviewer #3: Objective:

The authors aimed to identify various bacterial pathogens in ticks in different ecological settings of Inner Mongolia.

Overall, the authors obtained a substantial amount of data over the duration of 4 years from various locations across Mongolia. These could complement the existing literature on co-infections in other regions (for example : A study in France done by Moutailler et al. 2016. “Co-infection of Ticks: The Rule Rather Than the Exception”).

Is the study design appropriate to address the stated objectives:

The samples were taken from life stock, yet the introduction and discussion only refer to co-infections in human patients. I am assuming the authors are using the results to extrapolate them to humans, however, the authors did not discuss the implications (for example those tick species would need to also affect humans) and potential limitations for this (different biting rates, level of co-infections, how could the result differ depending on the host organism?). This to me is a discrepancy between the objective and study design.

The population was clearly described.

Is the sample size sufficient:

It is stated that sample area 1 has the highest tick infections and coinfections. However, sample area 1 has also by far the highest tick numbers collected. This could potentially attribute to a more likely detection of infection and co-infections (sample effect) compared to for example sample area 2.

Statistical analysis:

Chi2 ok.

ethical requirements:

The authors mentioned that consent for animal owners was verbally obtained. However, it is not clear whether/how this information was recorded.

**Response:** Thanks for the reviewer’s comments, in this study was to investigate the distribution characteristics of ticks, and the prevalence, co-infection rates and diversity of tick-borne pathogens in questing ticks collected from three sites different ecological in Inner Mongolia. Although the number of samples in the above three regions is not completely the same, there may be sample effect, but this study mainly analyzed the proportion of infection. The results showed that the infection rate of pathogens and the degree of co-infection were different in three sites different ecological in Inner Mongolia, which suggested that it would have an impact on clinical diagnosis.

About the consent for the animal owners, ticks in areas 2 and 3 were collected from the animals, while animals were domestic animals (cattle, horses, sheep and camels). All the sampling was done with the consent of the owner and the help of the owner. Although the consent form was not signed, there were some photos and videos documenting the cooperation of animal owners during tick collection.

--------------------

**Results**

Does the analysis presented match the analysis plan?

-Are the results clearly and completely presented?

-Are the figures (Tables, Images) of sufficient quality for clarity?

Reviewer #1: (No Response)

Reviewer #2: -Please verify whether only adult ticks analysed (or were there any immatures included).

-What are the differences observed between male and female ticks in terms of infection and co-infections. Please discuss.

**Response:**

**(1)** Only adult ticks were included in the study.

**(2)** The infection rate of male ticks was higher than that of female ticks, and the co-infection rate of female ticks was higher than that of male ticks. By region, the infection rate is generally higher in males than in females, at the co-infection rate, the females are higher than the males in region 1, and the differences between regions 2 and 3 are not obvious. The differences or regularites observed between male and female ticks were not obvious in terms of infection and co-infections.

Reviewer #3: The manuscript suffers from poor grammar throughout the manuscript to the point where sentences become unreadable, and the meaning gets lost.

The results are clearly presented in the tables, however there is a discrepancy between the results and the discussion.

In the results section the overall infection rate is 61.4%, in the discussion it is 69.8%.

The figures and tables are of sufficient clarity.

**Response:** In response to the reviewer’s comment, we have asked a professional team to modify the language and then submitted the revised manuscript. In addition, we double-checked and corrected the inconsistencies between the results and the data in the discussion.

--------------------

**Conclusions**

-Are the conclusions supported by the data presented?

-Are the limitations of analysis clearly described?

-Do the authors discuss how these data can be helpful to advance our understanding of the topic under study?

-Is public health relevance addressed?

Reviewer #1: (No Response)

Reviewer #2: -Please explain more on how clinicians can used the information for the purpose of diagnosis and treatment. Is there a need for them to keep the ticks for species identification?

-Are the tick-borne diseases can be treated in a similar manner or using similar drugs?

-What about the clinical presentations between the different types of pathogens? Any particular sign or symptoms to look for?

**Response:** Different tick species carry different kinds of pathogens, and these theories can be seen in several published papers. And the disease caused by different pathogens is completely different, so the typical clinical symptoms are different, the severity of the disease is also very different. For example, the main vector of forest encephalitis is *I. persulcatus*, which can cause sudden high fever, headache, and disturbance of consciousness. Rickettsial disease is mainly transmitted by *Dermacentor* and *Haemaphysalis*, which can cause symptoms such as rash, headache, and slow reaction. Therefore, clinicians can assist in diagnosis based on tick species identification.

Tick-borne diseases with no response to antivirals or most antibiotics, but tetracyclines are exception. Use early to avoid complications. However, due to the lack of typical clinical manifestations and laboratory techniques, it is easy to be misdiagnosed and develop multiple organ failure leading to death. Co-infections is important as they do not just present diagnostic challenges, but the pathogens might also exacerbate the disease.

Reviewer #3: Are the conclusions supported by the data presented/ are there limitations:

Partially, as mentioned before it would be interesting to know whether those tick species found on cattle are the same ones that infect humans.

Further, it would be also interesting to know whether there was a change in the number of tick species/ coinfections over time (the study period was 4 years) and if yes, what impact this could have.

**Response:** The above conclusion can be basically supported. As for the time factor, it's certainly there, but the change over a short period of time is not very obvious. And due to the wide collection span and limited peak tick activity, it is not possible to ensure the same collection area every year, so it is not possible to estimate the influence of time factors.

**Understanding of the topic and impact on public health:**

**The authors found a high infection rate and coinfection rates in particular in one sample area, which consists mainly of forest areas. The authors did mention that knowledge of co-infections is important as they do not just present diagnostic challenges, but the pathogens might play different roles within their respective hosts, thus modulating disease severity.**

--------------------

**Editorial and Data Presentation Modifications?**

Use this section for editorial suggestions as well as relatively minor modifications of existing data that would enhance clarity. If the only modifications needed are minor and/or editorial, you may wish to recommend “Minor Revision” or “Accept”.

Reviewer #1: (No Response)

Reviewer #2: It is better for the manuscript to be checked for typing and spacing errors. A proofread by a native speaker may be useful too.

Reviewer #3: (No Response)

**Response:** In response to the reviewer’s comment, we have checked for typing and spacing errors. In addition, we have asked a professional team to correct the language before submitting the revised manuscript this time.

--------------------

**Summary and General Comments**

Use this section to provide overall comments, discuss strengths/weaknesses of the study, novelty, significance, general execution and scholarship. You may also include additional comments for the author, including concerns about dual publication, research ethics, or publication ethics. If requesting major revision, please articulate the new experiments that are needed.

Reviewer #1: Liu et al analyzed a mount of ticks samples collected from vegetation or animals.

Main comments.

1, ticks from vegetation and animals should be analyzed separately.

**Response:** Ticks collected from area 1 were collected from vegetation in the forest, ticks collected from area 2 and area 3 were all collected from animal body surface. The data in this paper are respectively analyzed the infection rate and co-infection rate of three areas (forest, grassland and Gobi desert).

2, The result of co-infection was so confusing. please analyze it by Multivariate analysis.

**Response:** In this study was to describe the regional differences in co-infection outcomes, and found that pathogen diversity varied with ecogeographic differences such as gobi desert, grassland and forest. In addition, some samples were collected on timescales longer and involved a wide range, and some collection sites did not record other detailed data except ecological characteristics, there are certain difficulties in multivariate analysis at present. In future studies, we will regression tick collection sites, record detailed variable factors that may affect tick distribution and infection, and then analyze them.

3, Please re-write the purpose of introduction. Because authors did not analyze the genotype diversity in this MS.

**Response:** We have modified it. We wanted to express the diversity of tick-borne pathogens and co-infection types.

Minor comment

1, Title would be better to change, for focusing the co-infection.

2, Please add more information of each detected pathogens

3, please indicate the tick stages

**Response:** In response to the reviewer’s comment, we have added the message of tick stage in the first sentence of results. And the title is changed to “Diversity and co-infection of tick-borne bacterial pathogens in ticks collected in Inner Mongolia”. The each detected pathogens are listed in the article and table.

Reviewer #2: Great and interesting findings.

**Response:** Thank you for your affirmation.

Reviewer #3: Summary:

In this manuscript, the authors identified various bacterial pathogens in ticks in different ecological settings of Inner Mongolia. They found a high infection rate and coinfection rates in particular in one sample area, which consists mainly of forest areas. Knowledge of co-infections is important as they do not just present diagnostic challenges, but the pathogens might play different roles within their respective hosts, thus modulating disease severity.

Overall, the authors obtained a substantial amount of data over the duration of 4 years from various locations across Mongolia. These could complement the existing literature on co-infections in other regions (for example: A study in France done by Moutailler et al. 2016. “Co-infection of Ticks: The Rule Rather Than the Exception”). However, unfortunately, the manuscript suffers from poor grammar throughout the manuscript to the point where sentences become unreadable, and the meaning gets lost.

Therefore, unless the grammar is significantly improved I do not recommend the manuscript for publication.

**Response:** First of all, thank you for your affirmation of the research results. we have asked a professional team to correct the language before submitting the revised manuscript this time.

Further general comments:

1. Extrapolation of results:

The samples were taken from life stock, yet the introduction and discussion only refer to co-infections in human patients. I am assuming the authors are using the results to extrapolate them to humans, however, the authors did not discuss the implications (for example those tick species would need to also affect humans) and potential limitations for this (different biting rates, level of coinfections, how could the result differ depending on the host organism?).

**Response:** In response to the reviewer’s comment, regarding the survey results of this paper: (1) A variety of tick-borne bacterial pathogens were identified, with an overall high prevalence rate (61.4% of ticks infected). (2) The frequency of co-infection: Among infected ticks, 24.2% were co-infected. There are many pathogens and co-infections carried by ticks, suggesting a high potential threat to humans.

In this study also to describe the regional differences in infection and co-infection rates outcomes, and found that pathogen diversity varied with ecogeographic differences such as gobi desert, grassland and forest. Geographical differences affect tick species and tick-borne pathogens, so the potential threat to humans or animals is also different.

2. Discrepancy between results and discussion:

In the results section the overall infection rate is 61.4%, in the discussion it is 69.8%.

**Response:** In response to the reviewer’s comment, we have double-checked and corrected it.

3. As mentioned the overall grammar is poor (switch between past and present tense, singular and plural). Examples include:

Line 47-49- “Tick borne pathogen is transmitted from hematophagous ticks, and the tick sometimes harbored multiple pathogens, blood sucking host (including human) is infectious to pathogens”

Line 49- “When tick carries plural kinds of pathogen, co-infection often occurred.”

Line 55- At the same time, which greatly increases the probability of co-infection of host animals and humans, leading to the epidemic of tick-borne diseases

**Response:** In response to the reviewer’s comment, we have asked a professional team to correct the language before submitting the revised manuscript this time. .

4. The language is informal and below publication standard for a Scientific Journal (for example authors are named by first name in Line 63 “Antonia”)

**Response:** In response to the reviewer’s comment, we have modified and corrected the language.

5. Simple errors were made that look like the paper was not proof-red – for example lack of spacing “Prevalence of tick-borne pathogens in ticks”

**Response:** In response to the reviewer’s comment, we have corrected it. Different versions of office software are inconsistent, which may cause confusion during transmission. We have checked and corrected the full text.

6. In the abstract it is highlighted first what could not be shown before the actual results are presented:

“Due to the lack of epidemiological data and specific clinical symptoms related to co-infection of tick-borne pathogens, we couldn't accurately and rapidly distinguish between a single pathogen infection and multiple pathogens co-infection, resulting in negligence of co-infection and even serious consequences. In our study, we summarized the possible co-infection types and the differences of coinfection among different ecological regions through the tick collection, experiment, sequencing, comparison and analysis of tick samples collected from all Inner Mongolia.”

**Response:** In response to the reviewer’s comment, some of the problems highlighted in this abstract are factual. The lack of accurate and rapid diagnostic techniques in the clinic has led to the prevalence of undetected fever (unknown fever). Therefore, we first conducted a survey on the vector borne, because ticks are abundant in Inner Mongolia.

7. It is stated that area 1 has the highest tick infections and coinfections. However, sample area 1 has also by far the highest tick numbers collected, could this not also attribute to a more likely detection of infection and co-infections (sample effect?) compared to for example sample area 2?

**Response:** Thanks for the reviewer’s comments, in this study was to investigate the distribution characteristics of ticks, and the prevalence, co-infection rates and diversity of tick-borne pathogens in questing ticks collected from three sites different ecological in Inner Mongolia. Although the number of samples in the above three regions is not completely the same, there may be sample effect, but this study mainly analyzed the proportion of infection. The results showed that the infection rate of pathogens and the degree of co-infection were different in three sites different ecological in Inner Mongolia, which suggested that it would have an impact on clinical diagnosis.

Other specific comments:

Line 51- Why are these regional differences so “obvious”? Delete the word “obvious”.

**Response:** We have deleted the word “obvious”.

Line 67- This assumes the reader knows that *Candidatus* Rickettsia tarasevichiae is transmitted via ticks.

**Response:** *Candidatus* Rickettsia tarasevichiae (CRT) was initially detected in *Ixodes persulcatus* ticks in Russia, human infection with CRT was first reported in northeastern China in 2012.

Line 87- 81- I do not understand the authors argument here. Do you mean that the focus has been too much on one type of tick-borne infection?

**Response:** What I'm trying to say is that most studies have focused on co-infection of pathogens type, not genotypes. We have changed the corresponding description.

Line 83- What are superficial co-infections and how does it differ from your work? Is it because you are collecting data from 3 different sites?

**Response:** It was my inaccurate expression that caused misunderstanding. We have made modifications in the text. Indeed, most studies have focused on the identification of diversity in a single or a few kind of pathogens, as well as the prevalence of pathogen species.

Line 98-99- ample sunshine is not a scientific term

**Response:** We have deleted the corresponding description, and asked a professional team to correct the language before submitting the revised manuscript this time.

Line 155- How many/ what tick species are there?

**Response:** A total of eight tick species were collected, and the corresponding descriptions are given in the first sentence of the results section.

Line 140-148- Why did you need to clone the PCR products into plasmids first before sequencing? Was the yield too low?

**Response:** The *p44/msp2* and *p28/omp-1* genes were used for the detection of *Anaplasma* and *Ehrlichia*. The *p44* and *p28* are both multigene family, with 113 and 28 *p44/msp2* and *p28/omp-1* paralogous genes, including pseudogenes, are distributed throughout the genome and contain a hypervariable region flanked by 5` - and 3` - end conserved regions, respectively. Therefore, PCR products cannot be direct sequence, post-sequencing of TA-cloning is required.

Line 169 – check the spacing of words

**Response:** Different versions of office software are inconsistent, which may cause confusion during transmission. We have checked and corrected the full text.

Line 188-191- Do you mean it could be any of those species and a further distinction is impossible?

**Response:** There are several types of rickettsia that we've detected in this study, *R. raoultii* (accession no. DQ365803), *Candidatus* R. tarasevichiae (accession no. MN450397), and *Rickettsia* sp. strain YN02 (accession no. KY411135).

Line 188-198- It looks like the analysis was done for particular genes that identify a particular set of species. However, what is that selection based on? Are those genes only present in those particular species or is it based on percentage of sequence identity? Can you combine the scores for various genes to narrow down the potential infected species?

**Response:** These particular genes used in the detection are all genes with high specificity for tick-borne pathogens and have been fully confirmed in previous studies. The *gltA* and *rOmpA* are specific gene for SFGR in *Rickettsia*, which can detect all spotted fever group rickettsia (SFGR). The *p44/msp2* and *p28/omp-1* are specific genes for *Anaplasma* and *Ehrlichia* respectively, which can be used to detect all of species of *Anaplasma* genus and *Ehrlichia* genus respectively. Th*e flaB* and *glpQ* are specific for Lyme disease spirochetes and relapsing fever spirochetes, respectively. The 16S rRNA is a multiplex real-time PCR assay for Lyme disease spirochetes and relapsing fever spirochetes.

Line 253- this study was about lifestock ticks, so it may be good to adjust this sentence accordingly

**Response:** We have modified. Co-infection will occur when a lifestock tick carrying multiple pathogens or multiple ticks carrying multiple pathogens bites a person in succession.

Line 259- in the results section the overall infection rate is 61.4% rather than 69.8%.

**Response:** We have corrected.

Line 262- Do you have an explanation of the high infection rate? Also the infection rate is higher than sample area 2 and 3, but is it high compared to other areas that have previously been studied?

**Response:** The whole area of Inner Mongolia is large, and we spent more than 5 years sampling. The infection rate and co-infection rate at the whole area level are also analyzed for the first time.

Line 267- This argument needs to be expanded. Why is this a threat? For whom (livestock, humans or both?) Is it meant to get worse? Data were collected over 4 years, did the authors see a change in time?

**Response:** We found a high infection rate and co-infection rates in particular in one sample area, which consists mainly of forest areas. We did mention that knowledge of co-infections is important as they do not just present diagnostic challenges, but the pathogens might play different roles within their respective hosts, thus modulating disease severity.

About the time factor, it's certainly there, but the change over a short period of time is not very obvious. And due to the wide collection span and limited peak tick activity, it is not possible to ensure the same collection area every year, so it is not possible to estimate the influence of time factors.

Line 307- specify the disease

**Response:** Co-infection of Lyme disease and rickettsial disease

Line 329- and lifestock?

**Response:** Added and modified livestock.

Line 551-552- The title of Tables 3 and 4 is more or less the same.

**Response:** Let's change the title of Table 3 to “Various tick-borne pathogens found in tick samples”.

--------

Figure Files:

While revising your submission, please upload your figure files to the Preflight Analysis and Conversion Engine (PACE) digital diagnostic tool, https://pacev2.apexcovantage.com. PACE helps ensure that figures meet PLOS requirements. To use PACE, you must first register as a user. Then, login and navigate to the UPLOAD tab, where you will find detailed instructions on how to use the tool. If you encounter any issues or have any questions when using PACE, please email us at figures@plos.org.

Data Requirements:

Please note that, as a condition of publication, PLOS' data policy requires that you make available all data used to draw the conclusions outlined in your manuscript. Data must be deposited in an appropriate repository, included within the body of the manuscript, or uploaded as supporting information. This includes all numerical values that were used to generate graphs, histograms etc.. For an example see here: http://www.plosbiology.org/article/info%3Adoi%2F10.1371%2Fjournal.pbio.1001908#s5.

Reproducibility:

To enhance the reproducibility of your results, we recommend that you deposit your laboratory protocols in protocols.io, where a protocol can be assigned its own identifier (DOI) such that it can be cited independently in the future. Additionally, PLOS ONE offers an option to publish peer-reviewed clinical study protocols. Read more information on sharing protocols at https://plos.org/protocols?utm_medium=editorial-email&utm_source=authorletters&utm_campaign=protocols

**Response:** In response to the reviewer’s comment, we have added these sequence data in Figure 2.

This revised manuscript has been reviewed and edited by native speaker before re-submitting.
