# Supplementary material for: Co-infection of tick-borne bacterial pathogens in ticks in Inner Mongolia, China
Source: PLoS Negl Trop Dis. 2023 Mar 9;17(3):e0011121. doi: 10.1371/journal.pntd.0011121 (PMC10030021; doi:10.1371/journal.pntd.0011121)
Supplement: Attachment — Submitted filename: Response to Reviewers.docx [file pntd.0011121.s002.docx]

**Reviewer reports:**

Reviewer #2: Please state your reference for taxonomic keys/identification of the ticks using morphological characteristics.

Please state in the manuscript the types of livestock as you responded in your responses to reviewers/editor (i.e. cattle, goat, sheep, camel and horse).

Tick species were identified by morphological characteristics combined with tick *mt-rrs* gene identification method. The morphology of ticks is mainly based on experience, and there is no corresponding reference at present. We finally classified them by molecular identification method, the reference marked in paper.

About the types of livestock added in the manuscript.

[Ref 17] Construction of a DNA database for ticks collected in Japan: application of molecular identification based on the mitochondrial 16S rDNA gene. Medical Entomology and Zoology. 65(1); 13-21 (2014)。

Reviewer #3: It should be made clear in the text that ticks collected from area 1 were collected from vegetation in the forest,

ticks collected from area 2 and area 3 were all collected from animal body surface. This is not stated in the methods section.

We have added these instructions in the methods section.

--------------------

Results

-Does the analysis presented match the analysis plan?

-Are the results clearly and completely presented?

-Are the figures (Tables, Images) of sufficient quality for clarity?

Reviewer #2: (No Response)

Reviewer #3: The overall infection rates have been corrected and both the results and discussion now state the 61.4% overall infection rate.

However, I am not sure that referring to the overall co-infection rate of 24.2% is that useful because of the large variation between the three sample areas. I also agree with the other reviewer that those results from sample area 1 should be regarded separately from sample area 2 and 3 due to the different collection method. Co-infection rates in sample area 2 and 3 could be for example lower because they are collected on livestock (which is a fairly homogenous group, so exposure to other hosts might be limited). In the forest ticks were collected in the vegetation, these ticks may encounter different types of hosts much more thus they are more exposed to other pathogens).

We agree with the Reviewer 3, so in results of the co-infection, we adjusted the “total co-infection rate of 24.2%” to “the co-infection rate of the 3 regions was significantly different”. Then introduced the co-infection rates in regions 1, 2 and 3 respectively.

We described the ecology of regions 1, 2 and 3 in the paper, in sampling area 1, a forested area in the north-eastern part of Inner Mongolia, area 2 a grassland area in central Inner Mongolia, area 3 is the Gobi Desert and the semi-desertification steppe of the western part of Inner Mongolia. Ticks can only be collected by animal body surface because there is no tall grass in area 2 and 3, not use the same method as in area 1. From west to east in Inner Mongolia, the ecological environment is more better the further east, there's more diversity of pathogens.

--------------------

Conclusions

-Are the conclusions supported by the data presented?

-Are the limitations of analysis clearly described?

-Do the authors discuss how these data can be helpful to advance our understanding of the topic under study?

-Is public health relevance addressed?

Reviewer #2: Line 358 - Can you provide some examples of "relevant departments" and state these in your manuscript.

After 5 years of sampling and surveillance, we have found that the actual situation of co-infections, especially in area 1, the situation of multiple pathogens carried by ticks superimposed is beyond people's previous understanding. We hope that relevant government departments can pay attention to strengthen clinical diagnosis and treatment screening in areas where epidemic diseases occur, so as to avoid co-infection leading to severe disease among patients.

Sorry, we don't understand what this question means.

Reviewer #3: See comments in the results

--------------------

Editorial and Data Presentation Modifications?

Use this section for editorial suggestions as well as relatively minor modifications of existing data that would enhance clarity. If the only modifications needed are minor and/or editorial, you may wish to recommend “Minor Revision” or “Accept”.

Reviewer #2: The map (figure 1) can be more clearer/sharper. I recommend minor revision.

The map has been revised.

Reviewer #3: Line 103 and Line 104 specify “it”

“it” means the collection of ticks from the body surface of cattle, goats, and horses in this study.

--------------------

Summary and General Comments

Use this section to provide overall comments, discuss strengths/weaknesses of the study, novelty, significance, general execution and scholarship. You may also include additional comments for the author, including concerns about dual publication, research ethics, or publication ethics. If requesting major revision, please articulate the new experiments that are needed.

Reviewer #2: (No Response)

Reviewer #3: I think the authors have improved the manuscript significantly and answered my previous comments sufficiently. However, more detailed information in the methods section about the tick sampling method in the different areas is needed.

Tick sampling method in the different areas have been added to the method.
