# Supplementary material for: Co-infection of tick-borne bacterial pathogens in ticks in Inner Mongolia, China
Source: PLoS Negl Trop Dis. 2023 Mar 9;17(3):e0011121. doi: 10.1371/journal.pntd.0011121 (PMC10030021; doi:10.1371/journal.pntd.0011121)
Supplement: Peer review history

# Peer review: Decision Letter 0

**DOI:** 10.1371/journal.pntd.0011121.r001

**Authors:** Peter J. Krause, Johan Van Weyenbergh

23 Aug 2022

Dear Dr. Gaowa,

Thank you very much for submitting your manuscript "Tick-borne bacterial pathogens in ticks collected in Inner Mongolia, China" for consideration at PLOS Neglected Tropical Diseases. As with all papers reviewed by the journal, your manuscript was reviewed by members of the editorial board and by several independent reviewers. In light of the reviews (below this email), we would like to invite the resubmission of a significantly-revised version that takes into account the reviewers' comments. 

We cannot make any decision about publication until we have seen the revised manuscript and your response to the reviewers' comments. Your revised manuscript is also likely to be sent to reviewers for further evaluation.

When you are ready to resubmit, please upload the following:

[1] A letter containing a detailed list of your responses to the review comments and a description of the changes you have made in the manuscript. Please note while forming your response, if your article is accepted, you may have the opportunity to make the peer review history publicly available. The record will include editor decision letters (with reviews) and your responses to reviewer comments. If eligible, we will contact you to opt in or out.

[2] Two versions of the revised manuscript: one with either highlights or tracked changes denoting where the text has been changed; the other a clean version (uploaded as the manuscript file). 

Important additional instructions are given below your reviewer comments.

Please prepare and submit your revised manuscript within 60 days. If you anticipate any delay, please let us know the expected resubmission date by replying to this email. Please note that revised manuscripts received after the 60-day due date may require evaluation and peer review similar to newly submitted manuscripts.

Thank you again for your submission. We hope that our editorial process has been constructive so far, and we welcome your feedback at any time. Please don't hesitate to contact us if you have any questions or comments.

Sincerely,

Johan Van Weyenbergh

Academic Editor

PLOS Neglected Tropical Diseases

Peter Krause

Section Editor

PLOS Neglected Tropical Diseases

***********************

Reviewer's Responses to Questions

**Key Review Criteria Required for Acceptance?**

As you describe the new analyses required for acceptance, please consider the following:

**Methods**

-Are the objectives of the study clearly articulated with a clear testable hypothesis stated?

-Is the study design appropriate to address the stated objectives?

-Is the population clearly described and appropriate for the hypothesis being tested?

-Is the sample size sufficient to ensure adequate power to address the hypothesis being tested?

-Were correct statistical analysis used to support conclusions?

-Are there concerns about ethical or regulatory requirements being met?

Reviewer #1: Multivariate analysis would be better to add for analysis of co-infection.

Reviewer #2: -Please verify that only verbal consent is needed/required from the animal owners (no written consent).

-Please state the types of livestock involved.

-Please elaborate on the "animal physical examination method".

-Please include the taxonomic keys used for tick species identification.

-Please clarify the reference(s) for PCR protocol/gene used in the study and cite accordingly.

-Re-check whether the procedure for sequence analysis has been described adequately/thoroughly.

Reviewer #3: Objective:

The authors aimed to identify various bacterial pathogens in ticks in different ecological settings of Inner Mongolia.

Overall, the authors obtained a substantial amount of data over the duration of 4 years from various locations across Mongolia. These could complement the existing literature on co-infections in other regions (for example : A study in France done by Moutailler et al. 2016. “Co-infection of Ticks: The Rule Rather Than the Exception”).

Is the study design appropriate to address the stated objectives:

The samples were taken from life stock, yet the introduction and discussion only refer to co-infections in human patients. I am assuming the authors are using the results to extrapolate them to humans, however, the authors did not discuss the implications (for example those tick species would need to also affect humans) and potential limitations for this (different biting rates, level of coinfections, how could the result differ depending on the host organism?). This to me is a discrepancy between the objective and study design.

The population was clearly described.

Is the sample size sufficient:

It is stated that sample area 1 has the highest tick infections and coinfections. However, sample area 1 has also by far the highest tick numbers collected. This could potentially attribute to a more likely detection of infection and co-infections (sample effect) compared to for example sample area 2.

Statistical analysis:

Chi2 ok. 

ethical requirements:

The authors mentioned that consent for animal owners was verbally obtained. However, it is not clear whether/how this information was recorded.

--------------------

**Results**

-Does the analysis presented match the analysis plan?

-Are the results clearly and completely presented?

-Are the figures (Tables, Images) of sufficient quality for clarity?

Reviewer #1: (No Response)

Reviewer #2: -Please verify whether only adult ticks analysed (or were there any immatures included).

-What are the differences observed between male and female ticks in terms of infection and co-infections. Please discuss.

Reviewer #3: The manuscript suffers from poor grammar throughout the manuscript to the point where sentences become unreadable, and the meaning gets lost.

The results are clearly presented in the tables, however there is a discrepancy between the results and the discussion.

In the results section the overall infection rate is 61.4%, in the discussion it is 69.8%.

The figures and tables are of sufficient clarity.

--------------------

**Conclusions**

-Are the conclusions supported by the data presented?

-Are the limitations of analysis clearly described?

-Do the authors discuss how these data can be helpful to advance our understanding of the topic under study?

-Is public health relevance addressed?

Reviewer #1: (No Response)

Reviewer #2: -Please explain more on how clinicians can used the information for the purpose of diagnosis and treatment. Is there a need for them to keep the ticks for species identification?

-Are the tick-borne diseases can be treated in a similar manner or using similar drugs?

-What about the clinical presentations between the different types of pathogens? Any particular sign or symptoms to look for?

Reviewer #3: Are the conclusions supported by the data presented/ are there limitations:

Partially, as mentioned before it would be interesting to know whether those tick species found on cattle are the same ones that infect humans.

Further, it would be also interesting to know whether there was a change in the number of tick species/ coinfections over time (the study period was 4 years) and if yes, what impact this could have.

Understanding of the topic and impact on public health:

The authors found a high infection rate and coinfection rates in particular in one sample area, which consists mainly of forest areas. The authors did mention that knowledge of co-infections is important as they do not just present diagnostic challenges, but the pathogens might play different roles within their respective hosts, thus modulating disease severity.

--------------------

**Editorial and Data Presentation Modifications?**

Use this section for editorial suggestions as well as relatively minor modifications of existing data that would enhance clarity. If the only modifications needed are minor and/or editorial, you may wish to recommend “Minor Revision” or “Accept”. 

Reviewer #1: (No Response)

Reviewer #2: It is better for the manuscript to be checked for typing and spacing errors. A proofread by a native speaker may be useful too.

Reviewer #3: (No Response)

--------------------

**Summary and General Comments**

Use this section to provide overall comments, discuss strengths/weaknesses of the study, novelty, significance, general execution and scholarship. You may also include additional comments for the author, including concerns about dual publication, research ethics, or publication ethics. If requesting major revision, please articulate the new experiments that are needed.

Reviewer #1: Liu et al analyzed a mount of ticks samples collected from vegetation or animals.

Main comments.

1, ticks from vegetation and animals should be analyzed separately. 

2, The result of co-infection was so confusing. please analyze it by Multivariate analysis.

3, Please re-write the purpose of introduction. Because authors did not analyze the genotype diversity in this MS.

Minor comment

1, Title would be better to change, for focusing the co-infection.

2, Please add more information of each detected pathogens

3, please indicate the tick stages

Reviewer #2: Great and interesting findings.

Reviewer #3: Summary:

In this manuscript, the authors identified various bacterial pathogens in ticks in different ecological settings of Inner Mongolia. They found a high infection rate and coinfection rates in particular in one sample area, which consists mainly of forest areas. Knowledge of co-infections is important as they do not just present diagnostic challenges, but the pathogens might play different roles within their respective hosts, thus modulating disease severity.

Overall, the authors obtained a substantial amount of data over the duration of 4 years from various locations across Mongolia. These could complement the existing literature on co-infections in other regions (for example: A study in France done by Moutailler et al. 2016. “Co-infection of Ticks: The Rule Rather Than the Exception”). However, unfortunately, the manuscript suffers from poor grammar throughout the manuscript to the point where sentences become unreadable, and the meaning gets lost.

Therefore, unless the grammar is significantly improved I do not recommend the manuscript for publication. 

Further general comments:

1. Extrapolation of results:

The samples were taken from life stock, yet the introduction and discussion only refer to co-infections in human patients. I am assuming the authors are using the results to extrapolate them to humans, however, the authors did not discuss the implications (for example those tick species would need to also affect humans) and potential limitations for this (different biting rates, level of coinfections, how could the result differ depending on the host organism?).

2. Discrepancy between results and discussion:

In the results section the overall infection rate is 61.4%, in the discussion it is 69.8%.

3. As mentioned the overall grammar is poor (switch between past and present tense, singular and plural). Examples include:

Line 47-49- “Tick borne pathogen is transmitted from hematophagous ticks, and the tick sometimes harbored multiple pathogens, blood sucking host (including human) is infectious to pathogens”

Line 49- “When tick carries plural kinds of pathogen, co-infection often occurred.”

Line 55- At the same time, which greatly increases the probability of co-infection of host animals and humans, leading to the epidemic of tick-borne diseases

4. The language is informal and below publication standard for a Scientific Journal (for example authors are named by first name in Line 63 “Antonia”)

5. Simple errors were made that look like the paper was not proof-red – for example lack of spacing “Prevalenceof tick-borne pathogens inticks”

6. In the abstract it is highlighted first what could not be shown before the actual results are presented:

“Due to the lack of epidemiological data and specific clinical symptoms related to co-infection of tick-borne pathogens, we couldn't accurately and rapidly distinguish between a single pathogen infection and multiple pathogens co-infection, resulting in negligence of co-infection and even serious consequences. In our study, we summarized the possible co-infection types and the differences of coinfection among different ecological regions through the tick collection, experiment, sequencing, comparison and analysis of tick samples collected from all Inner Mongolia.”

7. It is stated that area 1 has the highest tick infections and coinfections. However, sample area 1 has also by far the highest tick numbers collected, could this not also attribute to a more likely detection of infection and co-infections (sample effect?) compared to for example sample area 2? 

Other specific comments:

Line 51- Why are these regional differences so “obvious”? Delete the word “obvious”.

Line 62 – What are those other tick-borne pathogens if they are not B. burgdorferi and Babesia?

Line 67- This assumes the reader knows that Candidatus Rickettsia tarasevichiae is transmitted via ticks.

Line 87- 81- I do not understand the authors argument here. Do you mean that the focus has been too much on one type of tick-borne infection? 

Line 83- What are superficial co-infections and how does it differ from your work? Is it because you are collecting data from 3 different sites?

Line 98-99- ample sunshine is not a scientific term

Line 155- How many/ what tick species are there?

Line 140-148- Why did you need to clone the PCR products into plasmids first before sequencing? Was the yield too low?

Line 169 – check the spacing of words

Line 188-191- Do you mean it could be any of those species and a further distinction is impossible?

Line 188-198- It looks like the analysis was done for particular genes that identify a particular set of species. However, what is that selection based on? Are those genes only present in those particular species or is it based on percentage of sequence identity? Can you combine the scores for various genes to narrow down the potential infected species?

Line 253- this study was about lifestock ticks, so it may be good to adjust this sentence accordingly

Line 259- in the results section the overall infection rate is 61.4% rather than 69.8%.

Line 262- Do you have an explanation of the high infection rate? Also the infection rate is higher than sample area 2 and 3, but is it high compared to other areas that have previously been studied?

Line 267- This argument needs to be expanded. Why is this a threat? For whom (livestock, humans or both?) Is it meant to get worse? Data were collected over 4 years, did the authors see a change in time?

Line 307- specify the disease

Line 329- and lifestock?

Line 551-552- The title of Tables 3 and 4 is more or less the same.

--------

Figure Files:

While revising your submission, please upload your figure files to the Preflight Analysis and Conversion Engine (PACE) digital diagnostic tool, https://pacev2.apexcovantage.com. PACE helps ensure that figures meet PLOS requirements. To use PACE, you must first register as a user. Then, login and navigate to the UPLOAD tab, where you will find detailed instructions on how to use the tool. If you encounter any issues or have any questions when using PACE, please email us at figures@plos.org.

Data Requirements:

Please note that, as a condition of publication, PLOS' data policy requires that you make available all data used to draw the conclusions outlined in your manuscript. Data must be deposited in an appropriate repository, included within the body of the manuscript, or uploaded as supporting information. This includes all numerical values that were used to generate graphs, histograms etc.. For an example see here: http://www.plosbiology.org/article/info%3Adoi%2F10.1371%2Fjournal.pbio.1001908#s5.

Reproducibility:

To enhance the reproducibility of your results, we recommend that you deposit your laboratory protocols in protocols.io, where a protocol can be assigned its own identifier (DOI) such that it can be cited independently in the future. Additionally, PLOS ONE offers an option to publish peer-reviewed clinical study protocols. Read more information on sharing protocols at https://plos.org/protocols?utm_medium=editorial-email&utm_source=authorletters&utm_campaign=protocols

# Author comment: Author response to Decision Letter 0

**DOI:** 10.1371/journal.pntd.0011121.r002

28 Oct 2022

Attachment Submitted filename: Response to Reviewers.docx Click here for additional data file.

# Peer review: Decision Letter 1

**DOI:** 10.1371/journal.pntd.0011121.r003

**Authors:** Peter J. Krause, Johan Van Weyenbergh

27 Dec 2022

Dear Dr. _,

Thank you very much for submitting your manuscript "Co-infection of tick-borne bacterial pathogens in ticks in Inner Mongolia, China" for consideration at PLOS Neglected Tropical Diseases. As with all papers reviewed by the journal, your manuscript was reviewed by members of the editorial board and by several independent reviewers. The reviewers appreciated the attention to an important topic. Based on the reviews, we are likely to accept this manuscript for publication, providing that you modify the manuscript according to the review recommendations. 

Please prepare and submit your revised manuscript within 30 days. If you anticipate any delay, please let us know the expected resubmission date by replying to this email. 

When you are ready to resubmit, please upload the following:

[1] A letter containing a detailed list of your responses to all review comments, and a description of the changes you have made in the manuscript. 

Please note while forming your response, if your article is accepted, you may have the opportunity to make the peer review history publicly available. The record will include editor decision letters (with reviews) and your responses to reviewer comments. If eligible, we will contact you to opt in or out

[2] Two versions of the revised manuscript: one with either highlights or tracked changes denoting where the text has been changed; the other a clean version (uploaded as the manuscript file).

Important additional instructions are given below your reviewer comments. 

Thank you again for your submission to our journal. We hope that our editorial process has been constructive so far, and we welcome your feedback at any time. Please don't hesitate to contact us if you have any questions or comments.

Sincerely,

Peter J. Krause

Section Editor

PLOS Neglected Tropical Diseases

Peter Krause

Section Editor

PLOS Neglected Tropical Diseases

***********************

Reviewer's Responses to Questions

**Key Review Criteria Required for Acceptance?**

As you describe the new analyses required for acceptance, please consider the following:

**Methods**

-Are the objectives of the study clearly articulated with a clear testable hypothesis stated?

-Is the study design appropriate to address the stated objectives?

-Is the population clearly described and appropriate for the hypothesis being tested?

-Is the sample size sufficient to ensure adequate power to address the hypothesis being tested?

-Were correct statistical analysis used to support conclusions?

-Are there concerns about ethical or regulatory requirements being met?

Reviewer #2: Please state your reference for taxonomic keys/identification of the ticks using morphological characteristics.

Please state in the manuscript the types of livestock as you responded in your responses to reviewers/editor (i.e. cattle, goat, sheep, camel and horse).

Reviewer #3: It should be made clear in the text that ticks collected from area 1 were collected from vegetation in the forest,

ticks collected from area 2 and area 3 were all collected from animal body surface. This is not stated in the methods section.

--------------------

**Results**

-Does the analysis presented match the analysis plan?

-Are the results clearly and completely presented?

-Are the figures (Tables, Images) of sufficient quality for clarity?

Reviewer #2: (No Response)

Reviewer #3: The overall infection rates have been corrected and both the results and discussion now state the 61.4% overall infection rate. 

However, I am not sure that referring to the overall co-infection rate of 24.2% is that useful because of the large variation between the three sample areas. I also agree with the other reviewer that those results from sample area 1 should be regarded separately from sample area 2 and 3 due to the different collection method. Co-infection rates in sample area 2 and 3 could be for example lower because they are collected on livestock (which is a fairly homogenous group, so exposure to other hosts might be limited). In the forest ticks were collected in the vegetation, these ticks may encounter different types of hosts much more thus they are more exposed to other pathogens).

--------------------

**Conclusions**

-Are the conclusions supported by the data presented?

-Are the limitations of analysis clearly described?

-Do the authors discuss how these data can be helpful to advance our understanding of the topic under study?

-Is public health relevance addressed?

Reviewer #2: Line 358 - Can you provide some examples of "relevant departments" and state these in your manuscript.

Reviewer #3: See comments in the results

--------------------

**Editorial and Data Presentation Modifications?**

Use this section for editorial suggestions as well as relatively minor modifications of existing data that would enhance clarity. If the only modifications needed are minor and/or editorial, you may wish to recommend “Minor Revision” or “Accept”. 

Reviewer #2: The map (figure 1) can be more clearer/sharper. I recommend minor revision.

Reviewer #3: Line 103 and Line 104 specify “it”

--------------------

**Summary and General Comments**

Use this section to provide overall comments, discuss strengths/weaknesses of the study, novelty, significance, general execution and scholarship. You may also include additional comments for the author, including concerns about dual publication, research ethics, or publication ethics. If requesting major revision, please articulate the new experiments that are needed.

Reviewer #2: (No Response)

Reviewer #3: I think the authors have improved the manuscript significantly and answered my previous comments sufficiently. However, more detailed information in the methods section about the tick sampling method in the different areas is needed.

--------------------

PLOS authors have the option to publish the peer review history of their article (what does this mean?). If published, this will include your full peer review and any attached files.

If you choose “no”, your identity will remain anonymous but your review may still be made public.

**Do you want your identity to be public for this peer review?** For information about this choice, including consent withdrawal, please see our Privacy Policy.

Reviewer #2: Yes: Dr. Aishah Hani Azil

Reviewer #3: No

Figure Files:

While revising your submission, please upload your figure files to the Preflight Analysis and Conversion Engine (PACE) digital diagnostic tool, https://pacev2.apexcovantage.com. PACE helps ensure that figures meet PLOS requirements. To use PACE, you must first register as a user. Then, login and navigate to the UPLOAD tab, where you will find detailed instructions on how to use the tool. If you encounter any issues or have any questions when using PACE, please email us at figures@plos.org.

Data Requirements:

Please note that, as a condition of publication, PLOS' data policy requires that you make available all data used to draw the conclusions outlined in your manuscript. Data must be deposited in an appropriate repository, included within the body of the manuscript, or uploaded as supporting information. This includes all numerical values that were used to generate graphs, histograms etc.. For an example see here: http://www.plosbiology.org/article/info%3Adoi%2F10.1371%2Fjournal.pbio.1001908#s5.

Reproducibility:

To enhance the reproducibility of your results, we recommend that you deposit your laboratory protocols in protocols.io, where a protocol can be assigned its own identifier (DOI) such that it can be cited independently in the future. Additionally, PLOS ONE offers an option to publish peer-reviewed clinical study protocols. Read more information on sharing protocols at https://plos.org/protocols?utm_medium=editorial-email&utm_source=authorletters&utm_campaign=protocols

References

Please review your reference list to ensure that it is complete and correct. If you have cited papers that have been retracted, please include the rationale for doing so in the manuscript text, or remove these references and replace them with relevant current references. Any changes to the reference list should be mentioned in the rebuttal letter that accompanies your revised manuscript. If you need to cite a retracted article, indicate the article's retracted status in the References list and also include a citation and full reference for the retraction notice.

# Author comment: Author response to Decision Letter 1

**DOI:** 10.1371/journal.pntd.0011121.r004

2 Jan 2023

Attachment Submitted filename: Response to Reviewers.docx Click here for additional data file.

# Editor report: Decision Letter 2

**DOI:** 10.1371/journal.pntd.0011121.r005

**Authors:** Peter J. Krause, Johan Van Weyenbergh

27 Jan 2023

Dear Dr. _,

We are pleased to inform you that your manuscript 'Co-infection of tick-borne bacterial pathogens in ticks in Inner Mongolia, China' has been provisionally accepted for publication in PLOS Neglected Tropical Diseases.

Before your manuscript can be formally accepted you will need to complete some formatting changes, which you will receive in a follow up email. A member of our team will be in touch with a set of requests.

Please note that your manuscript will not be scheduled for publication until you have made the required changes, so a swift response is appreciated.

IMPORTANT: The editorial review process is now complete. PLOS will only permit corrections to spelling, formatting or significant scientific errors from this point onwards. Requests for major changes, or any which affect the scientific understanding of your work, will cause delays to the publication date of your manuscript.

Should you, your institution's press office or the journal office choose to press release your paper, you will automatically be opted out of early publication. We ask that you notify us now if you or your institution is planning to press release the article. All press must be co-ordinated with PLOS.

Thank you again for supporting Open Access publishing; we are looking forward to publishing your work in PLOS Neglected Tropical Diseases.

Best regards,

Peter J. Krause

Section Editor

PLOS Neglected Tropical Diseases

Peter Krause

Section Editor

PLOS Neglected Tropical Diseases

***********************************************************

# Editor report: Acceptance letter

**DOI:** 10.1371/journal.pntd.0011121.r006

**Authors:** Peter J. Krause, Johan Van Weyenbergh

3 Mar 2023

Dear Dr. _,

We are delighted to inform you that your manuscript, "Co-infection of tick-borne bacterial pathogens in ticks in Inner Mongolia, China," has been formally accepted for publication in PLOS Neglected Tropical Diseases.

We have now passed your article onto the PLOS Production Department who will complete the rest of the publication process. All authors will receive a confirmation email upon publication.

The corresponding author will soon be receiving a typeset proof for review, to ensure errors have not been introduced during production. Please review the PDF proof of your manuscript carefully, as this is the last chance to correct any scientific or type-setting errors. Please note that major changes, or those which affect the scientific understanding of the work, will likely cause delays to the publication date of your manuscript. Note: Proofs for Front Matter articles (Editorial, Viewpoint, Symposium, Review, etc...) are generated on a different schedule and may not be made available as quickly.

Soon after your final files are uploaded, the early version of your manuscript will be published online unless you opted out of this process. The date of the early version will be your article's publication date. The final article will be published to the same URL, and all versions of the paper will be accessible to readers.

Thank you again for supporting open-access publishing; we are looking forward to publishing your work in PLOS Neglected Tropical Diseases. 

Best regards,

Shaden Kamhawi

co-Editor-in-Chief

PLOS Neglected Tropical Diseases

Paul Brindley

co-Editor-in-Chief

PLOS Neglected Tropical Diseases
